# Influence of gender and parental migration on IYCF practices in 6–23-month-old tribal children in Banswara district, India: findings from the cross-sectional PANChSHEEEL study

**DOI:** 10.1186/s40795-021-00491-7

**Published:** 2022-01-27

**Authors:** Hanimi Reddy Modugu, Rajesh Khanna, Antaryami Dash, Logan Manikam, Priti Parikh, Lorna Benton, Sanjay Sharma, Neha Santwani, Susrita Roy, Hemant Chaturvedi, Satya Prakash Pattanaik, Marie-Carine Lall, Virendra Kumar Vijay, Monica Lakhanpaul

**Affiliations:** 1 International Center for Research on Women, 59 South Extension-II, New Delhi, India; 2 Save the Children, National Support Office, Gurugram, India; 3 Department of Epidemiology and Public Health, UCL Institute of Epidemiology and Health Care, University College London, London, UK; 4 UCL Engineering for International Development Centre, Bartlett School of Construction and Project Management, University College London, London, UK; 5 Population, Policy and Practice Department, UCL Great Ormond Street Institute of Child Health, University College London, London, UK; 6 Save the Children India, Jaipur, India; 7 Institute of Education, University College London, London, UK; 8 Indian Institute of Technology (IIT), Delhi, India; 9 Community Paediatrics, Whittington NHS Trust, London, UK

**Keywords:** Gender. Migration, Infant and Young child feeding practices, Complementary-feeding practices, Minimum acceptable diet, Dietary diversity, Minimum meal frequency

## Abstract

**Background:**

The interdisciplinary Participatory Approach for Nutrition in Children: Strengthening Health, Education, Engineering and Environment Linkages (*PANChSHEEEL*) study used a participatory approach to develop locally-feasible and tailored solutions to optimise Infant and Young Child Feeding (IYCF) practices at an individual, household, community, and environmental level. This paper aims to evaluate the influence of gender; migration; and Health, Education, Engineering and Environmental (HEEE) factors on IYCF practices, with the primary outcomes being three key complementary-feeding practices of Minimum Dietary Diversity (MDD), Minimum Meal Frequency (MMF) and Minimum Acceptable Diet (MAD).

**Methods:**

A cross-sectional survey of 325 households with children aged 6–23 months was conducted in nine purposively selected villages in two blocks of Banswara district, Rajasthan, India. A survey tool was developed, translated into the local language, pre-tested, and administered in a gender-sensitive manner. Data-collection processes were standardized to ensure quality measures. Association of the primary outcome with 27 variables was tested using a Chi-square test (Mantel-Haenszel method); backward stepwise regression analysis was conducted to assess the impact of effect modifiers (gender, parental migration).

**Results:**

Half of the surveyed children were of each gender, and fathers from half of the households were found to have migrated within the previous year to search for additional income. Parental literacy ranged from 60 to 70%. More than half of the households had access to milk-producing animals. Consumption of each of the seven food groups, eggs (4.7% vs 0.7%; *p* < 0.02), MDD (10.5% vs 3.2%; *p* < 0.02) and MAD (9.4% vs 2.6%; *p* < 0.02) were higher for boys than for girls. After controlling for contextual factors, a male child was 4.1 times more likely to get a diet with MDD and 3.8 times more likely to get a diet with MAD. A child from a non-migrant household was 2.0–2.1 times more likely to get a diet with MDD and MAD as compared to a child from a migrant household. However, this association was not found to be statistically significant after regression. Presence of milk-producing animals in households and consumption of milk/milk products by children in the previous 24 h were the other two strong predictors of MDD and MAD, although access to animal milk in the house did not translate to an increase in consumption of milk/milk products by a child.

**Conclusion:**

Gender discrimination in diet diversity and complementary-feeding practices starts early in childhood with boys having a distinct advantage over girls. In the case of parental migration, further research is required to establish if it has an adverse impact on feeding practices. Emphasis needs to be given to gender issues and other contextual factors when developing strategies to optimise complementary feeding practices.

**Trial registration:**

With UCL ethics [Ethics ID 4032/002] in United Kingdom and with Sigma IRB [10,025/IRB/D/17–18] in India.

**Supplementary Information:**

The online version contains supplementary material available at 10.1186/s40795-021-00491-7.

## Background

Appropriate feeding of 6–23-month-old children enhances their chance of survival and promotes optimal growth and cognitive development. It is recommended that infants should be breastfed within 1 hour of birth, breastfed exclusively for the first 6 months of life, and continue to be breastfed up to 2 years of age and beyond [1]. Starting at 6 months, breastfeeding should be combined with safe, age-appropriate feeding of solid, semi-solid and soft foods [1]. Although adopting optimal feeding practices is fundamental to a child’s survival, growth, and development, few children benefit from these practices as caregivers often lack practical support, the time to take care of children, one-to-one counselling, and access to the correct information. Malnutrition is the result of a complex interplay between household factors like poverty, maternal-health literacy, diarrhoea, cooking fuel, home environment, dietary practices, and hygiene. The prevalence of these influences is a result of multiple socio-ecological factors including resources, awareness, and cultural and behavioural practices [2, 3]. UNICEF acknowledges the role of different determinants (immediate, underlying, enabling) of maternal and child nutrition and their interconnectedness for improved outcomes [4].

Infant and Young Child Feeding (IYCF) practices are a set of recommended caregiver practices to ensure that infants and young children receive the nutrition and care they need for optimal child survival, growth and development. Addressing IYCF practices is vital to attain the Sustainable Development Goals (SDG’s) 2.1 (end hunger and ensure access by all people to safe, nutritious and sufficient food all year round) and 2.2 (end all forms of malnutrition), that target ending all forms of malnutrition by 2030 [5]. The World Health Organization (WHO) has proposed eight core indicators for assessing IYCF practices in population-based surveys, out of which three are key complementary-feeding indicators. To reach Minimum Dietary Diversity (MDD), children aged 6–23 months must have consumed something from 4 or more food groups the previous day, out of the standard 7 food groups. To achieve Minimum Meal Frequency (MMF), however, breastfed and non-breastfed children 6–23 months of age must have received solid, semi-solid, or soft foods the minimum number of times or more in a day. Without adequate dietary diversity and meal frequency, infants and young children are vulnerable to malnutrition especially stunting, micronutrient deficiencies, and increased morbidity and mortality. The Minimum Acceptable Diet (MAD) is a composite indicator which combines MDD and MMF and assesses the adequacy of a child’s diet based on its micronutrient adequacy and meal frequency during the previous 24 h. Any child whose diet meets the MDD and the MMF criteria is considered to have a MAD [6].

Poor nutrition during the first 2 years of life (commonly known as the critical window of opportunity [7]) has long term consequences. During this period, incidence of stunting is highest due to children having a high demand for nutrients. In addition to disease prevention strategies, complementary-feeding interventions are most effective in reducing malnutrition and promoting the growth and development of children [8]. Achieving universal coverage of optimal breastfeeding practices could prevent 13% of deaths in children less than 5 years of age, whilst appropriate complementary-feeding practices could result in an additional 6% reduction in under-five mortality [9]. In developing countries, breastfed children are at least six times more likely to survive in the early months than children who are not. Inadequate growth in children in low-income countries is generally the consequence of infectious diseases and low nutrient intake, particularly inadequate energy and protein intake, relative to nutritional requirements [10].

In India, there are various programmes and initiatives aimed at improving child nutrition with a particular focus on the ‘first 1000 days approach’, such as the Integrated Child Development Services, National Health Mission, Mid-Day Meal Scheme, Targeted Public Distribution System, and National Food Security Mission. Recently the Government of India also launched the National Nutrition Strategy (in 2017) and the National Nutrition Mission (in 2018). The National Nutrition Mission provides an updated strategic framework for action to improve nutritional outcomes by holistically addressing the multiple determinants of malnutrition, through cross-sectoral convergence and contextualized planning at each level of the implementation process [11].

Despite these initiatives, India’s performance in complementary-feeding practices is sub-optimal. This has been highlighted by the recent (2016–18) Comprehensive National Nutrition Survey (CNNS) for 6–23-month-old children, which has demonstrated that only 6% of children are receiving MAD (5.9% in breastfed Vs 7.6% in non-breastfed), 9% receiving iron-rich foods (8.0% in non-breastfed Vs 11.2% in breastfed), 21% meeting MDD (17.8% in non-breastfed Vs 35.6% in breastfed) and 42% meeting MMF (39.6 in breastfed Vs 50.0% in non-breastfed) [12]. The National Family Health Survey-4 (NFHS) [13] findings of 2015–16 also reported that less than one in ten children aged 6–23 months (9.6%) received an adequate diet. This figure includes 14.3% of non-breastfeeding children and 8.7% of breastfeeding children, with urban performance slightly better than rural. Comparison between NFHS-3 (2005–06) [14] and NFHS-4 (2015–16) also indicates that the complementary feeding rates have declined in India from 52.6% to 42.7%. Disaggregated data shows that there was intra-country variation in the feeding rate, with the highest decline observed in some of the southern states that have comparatively better-performing health systems [15].

There exists ample evidence of the influence of socio-economic inequalities, particularly economic status of the household on childhood malnutrition [16], but the evidence is limited on the impact of socio-economic variations on IYCF practices, particularly that of gender and parental migration. Within a household, food security and access to nutrition is closely related to decisions regarding responsibility for food production; earning cash; purchasing and preparation of food; and finally, access to food in terms of consumption [17]. In this regard, gender inequality plays an important role in skewing the distribution of food against the female child. Also, females are vulnerable to severe forms of malnutrition across all ages due to socio-cultural factors (responsible for childbearing and rearing, being the last one to consume food at mealtimes in the family). Undernourished girls grow up to become undernourished women who give birth to a new generation of undernourished children [3]. In households living in poverty, women and girls are particularly disadvantaged in their access to household resources, including food and nutrition [18]. Gaps in water and sanitation services often mean that women spend 1–2 h every day collecting water and/or walking to open fields, further reducing time available for food preparation. Poor Water, Sanitation and Hygiene (WASH) services also increase pathways for disease transmission, some of which can reduce the ability of children to absorb nutrients. There is also growing evidence of discrimination against females for IYCF practices from India, including both breastfeeding and complementary feeding. A secondary analysis of the national data shows that girls are breastfed for shorter periods than boys and consume less milk, with the discrimination seen particularly in second-born girls [19].

The association between parental migration for work and child nutrition is mixed. On one hand, decreased parental time within a household results in a negative effect on child nutrition since parental time is important for food preparation or making sure that the child eats food that is nutritious. On the other hand, the money earned by migrated parents can positively affect child nutrition, as any added income is likely to improve a child’s nutrition by relieving income constraints [20]. Cameron and Lim (2007) found that having a parent out of home for work has a negative effect on short-term child nutrition, while they also found that providing families with remittances of over $200 can help to lessen the negative effect on child nutrition [21]. In households with multiple migration cycles, left-behind children are gaining recognition as a new and unique vulnerable group facing the consequences of early-initiated, inadequate, and low-quality complementary feeding [22]. The growing number of male migrants and episodes of migration have led to feminization of agriculture and waged labour, with consequent challenges for childcare and feeding [15, 23]. Thus, there seems to be no conclusive empirical evidence on the direct association between parental migration and child nutrition and literature on the association between parental migration and IYCF practices is quite limited.

Over the last decade, several studies have highlighted the need for interdisciplinary research to examine the association between undernutrition in children under five and different factors operating at household, community, government or health-system levels [15, 24, 25]. We conducted one such study, the Participatory Approach for Nutrition in Children: Strengthening Health, Education, Engineering and Environment Linkages (*PANChSHEEEL*) study funded by the Medical Research Council, UK. The study was designed to: (i) explore health, education, engineering and environmental (HEEE) factors that influenced IYCF practices and (ii) develop a socio-culturally appropriate, tailored, innovative, and integrated cross-sector HEEE package to support optimal IYCF practices through a community-led participatory approach [26].

Disaggregated data from the NFHS-4 points to the state of Rajasthan as having one of the lowest IYCF indicators in the country (Table 1). Within the state, Banswara district is known to be a predominantly tribal area and has IYCF indicators worse than the state average and than many of the other 32 districts. It was for this reason that Banswara was chosen as the study site.

**Table 1:** IYCF Indicators in India, Rajasthan and Banswara district, 2015–16 (in %)

Indicators	India	Rajasthan	Banswara
Early initiation of breastfeeding	41.5	28.4	37.8
Exclusive breastfeeding under 6 months	54.9	58.2	57.1
Introduction of solid, semi-solid or soft foods (6–8) months	42.7	30.1	Not available
Complementary feeding – Minimum Acceptable Diet	9.6	3.4	0.8

While poor socio-economic status of women is associated with poor child nutritional outcomes, the presence of high outmigration of fathers leads to feminisation of agriculture and hence less time being available to attend to feeding their children, thereby compromising their children’s growth and nutrition. With this in mind, we conducted a study to assess the variations in Diet Diversity (DD) and IYCF practices according to gender of the child and parental migration, and to test the association between HEEE characteristics of the household with three key IYCF measures of MMF, MDD and MAD. The study, conducted as part of the PANChSHEEEL project, involved a cross-sectional survey of 325 households across nine villages in the two program blocks (Ghatol and Kushalgarh) in, Banswara district, Rajasthan, India, where a child or children aged 6–23-month-old was present. The specific study objectives were:

1. To understand the HEEE characteristics of the households, including gender; parental migration; access to local resources (e.g., water); sanitation; energy; health; and educational practices.
2. To measure DD in terms of food and food groups consumed by children aged 6–23 months in the previous 24 h and record their IYCF practices.
3. To estimate the influence of two effect-modifiers (gender of the child and parental migration) on the different outcome variables of DD and IYCF practices
4. To measure the strength of association between the three key complementary-feeding measures (MDD, MMF and MAD) and effect-modifiers, with and without adjustment for other HEEE and background characteristics.

## Methods

### Study area

The household survey was conducted in the two community-development blocks (henceforth referred as ‘blocks’) situated in Banswara district of Rajasthan state in India. The two blocks of Ghatol and Kushalgarh were purposively selected since they represented two divergent agroeconomic dimensions within one district. Out of 637 villages in these two blocks, five villages were from the canal area of Mahi river, Ghatol block and four villages from the non-canal area of Kushalgarh block, selected for the survey based on a set of inclusion and exclusion criteria [26].

### Study design

The PANChSHEEEL study used a mixed-method study design consisting of both qualitative and quantitative data-collection techniques. The study was driven by the ‘socio-ecological model’ which entails a multisectoral approach for operationalizing IYCF practices at different levels (individual, family, community, policy level) [27]. The study also engaged a multi-disciplinary approach for obtaining data on IYCF knowledge and practices and for establishing interlinkages between different HEEE factors and IYCF practices. The quantitative survey was conducted from January to March 2017, covering households with a child aged 0–23-months on the day of the survey. Details of the qualitative data-collection methods used by this study are published elsewhere [28].

### Sample size, participants and tools

The sample size of households for the PANChSHEEEL study was designed to provide project-level estimates on HEEE and IYCF indicators. Assuming a true value of an indicator at 50%, with a 5% margin of error at 95% confidence level and a non-response rate of 15%, the minimum sample required for the study was estimated as 445 households with a child aged 0–23 months.

All the households in the nine program villages where mothers had a child aged 0–23 months were included in the survey provided the child was 0–23 months old on the day of the survey, both the child and the child’s mother were physically present at home *(*de-facto*)*, their details were part of the household roster developed by frontline health workers (Anganwadi workers-AWWs and Accredited Social Health Activists or ASHAs), and informed verbal consent was provided by both the mother and the head of the household. Twins and households with more than one child under 2 years were treated as separate subjects. Children who were permanent residents of these nine villages but were not available in the village on the day of data collection were excluded.

Since the key outcome indicators of the present manuscript are the three complimentary feeding practices of MDD, MMF and MAD, the sample was limited to 325 children aged 6–23 months. The estimated power of 6–23 month old sample is 0.98 with p_0_ = 0.5 and p_1_ = 0.6, with 95% confidence interval (https://www.stat.ubc.ca/~rollin/stats/ssize/b1.html).

The list of lactating mothers maintained by the frontline health workers (AWWs and ASHAs) in the village formed the basis for identifying households where there was a mother of a 0–23-month-old child. The list taken from the frontline health workers was further validated during interviews with mothers using a *‘snow-balling technique’* to prepare the final household list. The primary respondent for obtaining all child-related information (including feeding practices) was the mother. Other information on socio-demographic characteristics and details on HEEE domains of the house were obtained from the head of the household (often male), identified by the investigator while collecting demographic profiles of the household members. The survey tool (Appendix 1) was developed by drawing on existing questions that had been tried and tested in other cross-sectional surveys in India [13, 14], but also contained study-specific questions (on education, water, sanitation, energy, solid waste management) developed and finalized by subject experts from the PANChSHEEEL team. This multi-disciplinary team comprised of academics (from UCL, IIT-D, JNU) who were subject experts, staff from Save the Children with expertise in field implementation, and study investigators from the community who facilitated the participation of the local community throughout the study process. For data on socio-economic status, a modified Kuppuswami scale was used [29] while questions on food and IYCF were taken from the WHO-IYCF tool [6]. The finalized tool was translated into the local regional languages of the area (Hindi and Wagdi), and pre-tested by the research team in one non-program village in each of the two blocks. Based on the feedback and learnings from the pre-testing, the questionnaire was revised with the addition/deletion of some questions and refinement of the language by replacing technical words with specific terms which were used more commonly in the local language. Four members of the research team (two male and two female investigators) and one supervisor were trained for 2 days followed by 1 day of field practice and de-briefing. The HEEE related sections of the tool were first administered by interviewing the head of the household by the male investigator, while IYCF related sections of the tool were administered later by the female investigator. All the investigators were conversant in both the local languages (‘Hindi’ and ‘Wagdi’) and had previous experience of data collection. Interviews were done in the local languages and investigators were sensitive to cultural gender differences. As the investigators belonged to the local community, they had good understanding of the local customs, practices, and perspectives which helped them to engage/communicate effectively with the respondents. The structure of the PANChSHEEEL team comprised of the main study team and the survey team, both teams working in close partnership to learn from each other, thereby strengthening the quality of data.

The survey was carried out during the lean agricultural season (January–March, 2017) to ensure the availability of the respondents at home. The field data-collection process was supervised by one full-time supervisor, and fortnightly meetings of all the investigators and the supervisor were held to review the coverage and quality of the data collected in the previous weeks by comparing sample completed questionnaires.

After verifying the list of lactating mothers with children aged 0–23 months provided by ASHA and AWW, the data-collection team successfully identified and interviewed 445 households with mothers with a child aged 0–23 months on the day of data collection. However, for the current manuscript we have confined our analysis only to 325 children as the focus of manuscript is to understand diet and IYCF practices among 6–23-month-old children.

The primary outcome for the program was IYCF indicators, however for this article we have taken the three complementary-feeding practices of MDD, MMF and MAD as key outcome indicators. A number of independent variables (HEEE, socio-economic and demographic) were first included in bivariate analysis to test their association with the three IYCF practices. Out of the 27 independent variables included in the initial analysis, gender and parental migration were identified as effect-modifiers and their association was then tested with the three IYCF practices (Additional file 2). To independently test the association of the two effect modifiers, stepwise logistic regression analysis was conducted using 14 other covariates. This analysis excluded the covariates that were highly skewed towards a single response and ensured that each of the HEEE domains was represented by at least one covariate. All IYCF indicators were estimated using the standard definitions [6].

### Statistical analysis

All analyses were done using the SPSS statistical software package Version 22 and Stata Version 14. Standard descriptive statistics are presented as percentages and means with standard deviations, while types of food consumed and IYCF factors are presented as percentages with 95% CI. The strength of association between the explanatory variables and the outcomes (MDD, MMF and MAD) was presented as an unadjusted Odds Ratios (OR) and tested using a Chi-square test (Mantel-Haenszel method) with a *p*-value of < 0.05 for significance testing. The two effect modifiers (gender and migration) were included in the regression model, while backward stepwise regression was used to exclude other factors which showed weak association. Adjusted OR results were only presented for the covariates that were part of the final regression model.

Standard ethical processes were followed, and formal ethical clearance was taken from the Ethics Committee of University College London UK, and from the Sigma-IRB in India. No one apart from the principal investigators (PI)/Co-PIs were given access to anonymised raw data, after removing personal identifiers.

## Results

### Socio-environmental profile

Out of a total of 445 households with a child aged 0–23-months covered by the survey, 325 households had a child aged 6–23 months, and the findings of this manuscript are confined to these children. Table 2 provides the HEEE and background characteristics of these 325 households. There were equal numbers of children from each block with nearly equal gender representation (52.6% boys vs. 47.6% girls). Parental migration (mostly the father of the child) for work in the past 1 year was nearly four times higher in the Kushalgarh block as compared to Ghatol block (74% vs. 19%), implying that migration-related differences in IYCF practices were mostly confined to Kushalgarh. Variation was observed in terms of access and utilization of different health-care services which are provided free of cost under the national programmes. While 95% of children had Mother and Child Protection (MCP) cards, only 57% of children aged 12–23 months were fully immunized *(received BCG, 3 doses of DPT, 3 doses of OPV and one dose of measles vaccine)*, 66% received Vitamin-A supplementation, and only 26% received deworming tablets during the past 6 months as part of the national deworming programme. Households were predominantly dependent on agriculture as their main source of income (88%); half of them had access to safe drinking water within the house *(from a protected water source like a tube well or covered well)*; one in five (19%) households had improved sanitation facilities *(*e.g.*, used toilet in the house and washed hands with soap)*; and fewer houses (7%) had clean cooking fuel *(liquid petroleum gas or solar energy)*. A large number of households (59–85%) had access to animal milk, with 85% of households having cow/bull/buffaloes, 60% having goats, and more than half (54%) having both types of animals. Three-quarters of the households were from the scheduled tribe (ST), around one-third (36%) had poor income *(< Rs 2020 per month)* and 62% of households had two or more children under the age of 5 years.

**Table 2:** Percentage distribution of background and HEEE characteristics of the households with 6–23 months old children, Banswara, Rajasthan, India

Health, Education, Engineering and Environment (HEEE) Characteristics	N - Valid cases	Frequency	% Frequency
**Gender of child**			
Boy	325	171	52.6
Girl	325	154	47.4
**Migration**			
In Ghatol block	166	32	**19.3**
In Kushalgarh block	159	117	**73.6**
Total	325	149	45.8
**Education**			
Literate head of the households	325	228	70.2
Literate caretaker of child (mother)	325	203	62.5
Head of the house aware of Swachh Bharat Campaign	325	195	60.0
**Health**			
**Access to AWC services and growth monitoring**			
Child going to AWC on a regular basis	325	241	74.2
Child being weighed in last 3 months	325	172	52.9
Child being weighed once every month	325	124	38.0
Immunization or MCP card available	204	194	95.0
**Immunization and Access to micronutrients in 12–23 month old children**			
Child was fully immunised	204	117	57.3
Child had Vit-A in last 6 months	204	135	66.2
Child had Albendazole in last 6 months	204	54	26.4
**Engineering and Environment**			
Cultivated kitchen garden inside house	325	14	4.3
Household having access to safe drinking water (own hand pump)	325	169	52.0
Household having improved fuel source for cooking	325	21	6.5
Toilet			
In use	325	70	21.5
Not in use or No toilet	325	255	78.5
Household having improved sanitation facility	325	61	18.8
**Access to milk and poultry products**			
Household with cows/bulls/buffaloes	325	275	84.6
Household with goats	325	193	59.4
Households with cows/bulls/buffaloes and goats	325	174	53.5
Households with access to poultry products	325	92	28.3
**Background characteristics**			
Block			
Ghatol	325	166	51.1
Kushalgarh	325	159	48.9
Hindu households	325	325	100.0
Caste			
Scheduled Tribe	325	239	73.5
Others	325	86	26.5
Monthly Income of family			
< = Rs 2020 (Poor)	325	118	36.3
> Rs 2020 (Middle/rich)	325	207	63.7
Households with agriculture as occupation	325	286	88.0
Age of mother (years)			
17–24	325	83	25.5
25–45	325	242	74.5
*Mean age (SD)*	325	*26.4 (5.6)*	
Number of children under 5 in household			
More than one	325	200	61.5
Only one	325	125	38.5

### Breastfeeding

Prevalence of breastfeeding-related IYCF practices was much higher compared to diet-related practices in children aged 6–23 months (Table 3). Breastfeeding was initiated within 1 hour of birth in nearly 60% of children and 94% were exclusively breastfed for the first 6 months, though few (8%) children were given pre-lacteal feeds immediately after birth. Breastfeeding was continued till 12–23 months of age, and in 80% of children aged more than a year. Semi-solid foods were introduced in only two-out-of-five children aged 6–8 months. While more than half of the children (55%) had access to meals more than 4 times a day, access to a diverse diet and adequate diet was very poor (MDD 7%, MAD 6%). Of the seven major food groups considered for dietary diversity, grains in any format were the most common food group consumed by the majority of children (70%) during the previous 24 h, followed by milk and dairy products (38%), and legumes and nuts (30%). Although 59–85% households had access to animal milk (Table 2), consumption of milk/dairy products by children was only 38% (95% CI: 33–44%). Consumption of eggs (3%) and vitamin-A-rich fruits and vegetables (6%) was very low, though around 12% of children had consumed other fruits and vegetables in the previous 24 h. Consumption of convenience foods (biscuits, wafers) and tea was high among the children (37 and 31% respectively), whilst a negligible number of children (1.5%) had consumed iron rich foods like meat and fish in the previous 24 h.

**Table 3:** Percentage prevalence of Diet Diversity^a^ and IYCF^b^ practices and Nutritional status of children aged 6–23 months, Banswara, Rajasthan, India

Type of Indicator	6–23 month old children		N (All valid cases)
	% Estimate	95% CI of estimate	
**Nature of specific food items/food groups child consumed in last 24 h**			
Grains	70.3	65.0–75.2	323
Legumes	30.4	25.5–35.8	323
Milk	38.4	33.1–44.0	323
Eggs	2.8	1.3–5.2	323
Vit-A rich fruits and vegetables	6.2	3.8–9.4	323
Other fruits and vegetables	11.9	8.5–15.9	323
Rice/Porridge	12.9	9.3–16.6	325
Tea	31.1	26.0–36.0	325
Biscuits/Wafers	37.2	32.0–42.5	325
Roti/Chapati	54.5	49.0–60.0	325
**Infant and Young Child Feeding (IYCF) practices**			
Early initiation of breastfeeding < 1 HR of delivery	58.5	52.9–63.9	325
Child was given pre-lacteal feeding	7.7	5.9–11.9	325
Exclusive breastfeeding for 6 months	94.2	91.6–96.7	325
Child was breastfed yesterday	85.2	80.9–88.9	325
Breastfeeding continued until 12–23 months	79.9	74.4–85.4	204
Age-appropriate breastfeeding (AABF)	67.1	61.9–72.9	325
Child had access to diet with minimum meal frequency (MMF)	54.5	48.9–59.9	325
Child had access to diet with minimum dietary diversity (MDD)	7.1	4.9–10.9	325
Child had access to food with minimum acceptable diet (MAD)	6.2	3.9–9.9	325
Child had iron-rich food yesterday	1.5	0.5–3.6	325

^a^Food items/groups consumed by child in past 24 h. ^b^Infant and Young Child Feeding

Table 4 presents the gender-based differentials of dietary and nutritional practices in terms of unadjusted ORs and Chi-square values. Consumption of each of the seven standard food groups was higher amongst boys than girls, although the difference was statistically significant only in relation to the consumption of eggs. On the contrary, consumption of food items such as rice, tea, biscuits and roti/chapati were higher among girls as compared to boys, but the difference was statistically significant only in the case of roti/chapatis. All the IYCF practices were higher amongst boys, but it was access to a diverse diet (3.5 times) and to an adequate diet (3.9 times) which were significantly higher for boys as compared to girls.

**Table 4:** Gender differentials in percentage prevalence and unadjusted odds ratios of food, IYCF and nutrition practices of children aged 6–23 months

Type of Indicator	% Prevalence		Unadjusted OR (95% CI of OR), Female as reference group	Chi-Square	***p***-value^*^
	In Girls (***n*** = 154)	In Boys (***n*** = 171)			
**Nature of specific food items/food groups child had in last 24 h**					
Grains	69.1	71.3	1.1 (0.7–1.8)	0.197	0.657
Legumes	28.9	31.8	1.1 (0.7–1.8)	0.300	0.584
Milk	34.9	41.5	1.3 (0.8–2.1)	1.471	0.225
Eggs	0.7	4.7	7.4 (0.9–59.6)	4.746	0.029
Flesh food	0.0	2.9	–	4.559	0.033
Vit-A rich fruits and vegetables	4.6	7.6	1.7 (0.7–4.4)	1.208	0.272
Other fruits and vegetables	8.7	14.6	1.8 (0.9–3.6)	2.636	0.104
**Solid, semi-solid, or soft foods consumed by child yesterday**					
Rice/Porridge	13.6	12.3	0.9 (0.5–1.7)	0.132	0.716
Tea	35.1	27.5	0.7 (0.4–1.1)	2.167	0.141
Biscuits/Wafers	39.0	35.7	0.9 (0.6–1.4)	0.374	0.541
Roti/Chapati	60.4	49.1	0.6 (0.4–1.0)	4.135	0.042
**Infant and Young Child Feeding (IYCF) practices**					
Early initiation of breastfeeding < 1 h of delivery	55.2	61.4	1.3 (0.8–2.0)	1.282	0.257
Child was given pre-lacteal feeding	6.5	8.8	1.4 (0.6–3.2)	0.591	0.442
Exclusive breastfeeding for 6 months	92.2	95.9	2.0 (0.8–5.2)	2.008	0.157
Child was breastfed yesterday	85.7	84.8	0.9 (0.5–1.7)	0.054	0.816
Breastfeeding continued till 12–23 months	79.6	80.2	1.0 (0.5–2.1)	0.012	0.914
Age-appropriate breastfeeding (AABF)	64.9	69.0	1.2 (0.8–1.9)	0.606	0.436
Complementary feeding introduced between 6 and 8 months	34.5	48.4	1.8 (0.6–5.0)	1.172	0.279

* *p*-value: Mantel-Haneszel

Table 5 provides parental migration-based differentials in dietary and IYCF practices. Among the standard food groups, consumption of milk, eggs, fruits, and vegetables were higher amongst non-migrant households than migrant households, but the difference was statistically significant only for milk consumption. Interestingly, consumption of popular food items such as rice, tea, biscuits and roti was higher amongst migrant households than their non-migrant counterparts, with consumption of roti/chapati being significantly higher among migrant households. There was not much difference between the proportion of children from migrant and non-migrant households who followed breastfeeding-related IYCF practices; however, the proportion of children following diet-related MDD & MAD practices was higher for non-migrant households, although the differences were statistically not significant. Initiation of complementary feeding between 6 and 8 months of age was significantly higher in non-migrant households than migrant households, while MMF was significantly higher in children from migrant households compared to non-migrant households.

**Table 5:** Migration based differentials in percentage prevalence and unadjusted odds ratios of food, IYCF, and nutrition practices of children aged 6–23 months

Type of Indicator	% Prevalence		Unadjusted OR (95% CI of OR), migrated as reference group	Chi-Square	***p***-value*
	Migrated (n-149)	Not migrated (***n*** = 176)			
**Nature of specific food items/food groups child had in last 24 h**					
Grains	71.6	69.1	0.9 (0.5–1.4)	0.235	0.628
Legumes	35.4	26.3	0.7 (0.4–1.1)	3.107	0.078
Milk	29.9	45.7	2.0 (1.2–3.1)	8.287	0.004
Eggs	2.0	3.4	1.7 (0.4–6.9)	0.565	0.452
Flesh food	2.0	1.1	0.6 (0.1–3.4)	0.408	0.523
Vitamin-A rich fruits and vegetables	3.4	8.6	2.7 (0.9–7.5)	3.654	0.056
Other fruits and vegetables	8.2	14.9	2.0 (1.0–4.0)	3.418	0.064
**Solid, semi-solid, or soft foods consumed by child yesterday**					
Rice/Porridge	14.8	11.4	0.7 (0.4–1.4)	0.827	0.363
Tea	36.2	26.7	0.6 (0.4–1.0)	3.416	0.065
Biscuits/Wafers	38.3	36.4	0.9 (0.6–1.4)	0.123	0.726
Roti/Chapati	66.4	44.3	0.4 (0.3–0.6)	15.877	0.000
**Infant and Young Child Feeding (IYCF) practices**					
Early initiation of breastfeeding < 1 h of delivery	48.3	67.0	2.0 (1.3–3.2)	9.102	0.003
Child was given pre-lacteal feeding	6.7	8.5	1.3 (0.6–3.0)	0.372	0.542
Exclusive breastfeeding for 6 months	96.0	92.6	0.5 (0.2–1.4)	1.649	0.199
Child was breastfed yesterday	85.9	84.7	0.9 (0.5–1.7)	0.099	0.753
Breastfeeding continued until 12–23 months	77.4	81.7	1.3 (0.7–2.6)	0.562	0.453
Age-appropriate breastfeeding (AABF)	64.4	69.3	1.2 (0.8–2.0)	0.871	0.351
Complementary feeding being introduced between 6 and 8 months	28.6	60.0	3.8 (1.3–11.1)	5.828	0.016

* *P* value - Mantel-Haneszel

Table 6 provides results from the stepwise logistic regression analysis to identify the association between the independent variables (HEEE, other background characteristics) with the three IYCF practices of MDD, MMF, and MAD, using gender and migration as the two effect-modifiers. After adjustment for HEEE and other background characteristics, a male child was found to be 4.1 times more likely to get a diet with MDD and 3.8 times more likely to get a diet with MAD as compared to a female child. Similarly, a child from non-migrant household was two times more likely to get a diet with MDD and MAD than a child from a migrant household, but this association did not reach statistical significance. Interestingly, MMF was found to be significantly higher among children from a migrant household compared to a non-migrant household. Among the HEEE and other background characteristics, higher literacy status of the head of the household, increased accessibility to milk-producing animals, and increased consumption of milk/milk products by the child in the previous 24 h were found to be strongest predictors of improved MDD, MMF, and MAD.

**Table 6:** Percent variations in prevalence of complementary feeding practices and strength of association of these practices in terms of adjusted and unadjusted OR, according to background and HEEE characteristics

HEEE and background Characteristics	MDD			MMF			MAD		
	% Children	Unadjusted OR (95% CI of OR)	Adjusted OR (95% CI of OR)^**$**^	% Children	Un-adjusted OR (95% CI of OR)	Adjusted OR (95% CI of OR)^**$**^	% Children	Unadjusted OR (95% CI of OR)	Adjusted OR (95% CI of OR)^**a**^
Sex of child									
Female	3.2	1.0	1.0	55.2	1.0	1.0	2.6	1.0	1.0
Male	10.5	3.5 (1.3–9.7)*	4.1 (1.3–12.`1)*	53.8	0.9 (0.6–1.5)	1.1 (0.7–1.8)	9.4	3.9 (1.2–11.8)*	3.8 (1.2–13.0)*
Any member of house migrated for work in the last year									
Migrated	4.7	1.0	1.0	61.7	1.0	1.0	4.0	1.0	1.0
Not migrated	9.1	2.0 (0.8–5.1)	1.9 (0.7–5.2)	48.3	0.6 (0.4–0.9)*	0.6 (0.4–1.0)*	8.0	2.1 (0.8–5.5)	2.0 (0.7–6.0)
Child going to AWC on a regular basis									
No	4.8	1.0	1.0	41.7	1.0	1.0	4.8	1.00	1.0
Yes	7.9	1.7 (0.6–5.2)	Removed in step-9	58.9	2.0 (1.2–3.3)**	2.1 (1.3–3.6)**	6.6	1.4 (0.5–4.4)	Removed in step-7
Literacy of head of household									
Illterate	1.0	1.0	1.0	51.5	1.0	1.0	1.0	1.0	1.0
Literate	9.6	10.3 (1.4–77.2)*	13.7 (1.6–115.2)*	55.7	1.2 (0.7–1.9)	Removed in step-6	8.3	8.7 (1.2–66.1)*	7.3 (0.9–58.2)
Literacy of caretaker of child (mother)									
Illiterate	10.7	1.0	1.0	49.2	1.0	1.0	9.0	1.0	1.0
Literate	4.9	0.4 (0.2–1.0)	Removed in step-6	57.6	1.5 (0.9–2.2)	Removed in step-12	4.4	0.5 (0.2–1.2)	Removed in step-10
Head of house aware of about Swachh Bharat Campaign									
No	3.8	1.0	1.0	56.2	1.0	1.0	2.3	1.0	1.0
Yes	9.2	2.5 (0.9–7.0)	Removed in step-11	53.3	0.9 (0.6–1.4)	Removed in step-8	8.7	4.1 (1.2–14.1)*	3.3 (0.9–12.9)
Household having access to safe drinking water in own house									
No	7.1	1.0	1.0	57.7	1.0	1.0	5.1	1.0	1.0
Yes	7.1	1.0 (0.4–2.4)	Removed in step-5	51.5	0.8 (0.5–1.2)	Removed in step-9	7.1	1.4 (0.6–3.6)	Removed in step-9
Household having improved fuel source for cooking									
No	6.3	1.0	1.0	54.9	1.0	1.0	5.9	1.0	1.0
Yes	19.0	3.5 (1.1–11.5)	5.1 (1.1–24.1)*	47.6	0.7 (0.4–1.8)	Removed in step-4	9.5	1.7 (0.4–7.7)	Removed in step-11
Household having improved sanitation facility									
No	6.4	1.0	1.0	54.2	1.0	1.0	5.3	1.0	1.0
Yes	9.8	1.6 (0.6–4.2)	Removed in step-10	55.7	1.1 (0.6–1.9)	Removed in step-5	9.8	2.0 (0.7–5.3)	Removed in step-6
Households with cows/bulls/buffaloes and goats									
No	4.0	1.0	1.0	47.7	1.0	1.0	2.6	1.0	1.0
Yes	9.8	2.6 (1.0–6.8)*	5.6 (1.7–18.5)**	60.3	1.6 (1.0–2.5)*	1.6 (1.0–2.5)	9.2	3.7 (1.2–11.4)*	5.8 (1.7–20.0)**
Block									
Kushalgarh	6.3	1.0	1.0	58.5	1.0	1.0	6.3	1.0	1.0
Ghatol	7.8	1.3 (0.5–3.0)	Removed in step-6	50.6	0.7 (0.5–1.1)	Removed in step-3	6.0	1.0 (0.4–2.4)	Removed in step-3
Caste									
Scheduled Tribe	7.9	1.0	1.0	56.5	1.0	1.0	7.5	1.0	1.0
Others	4.7	0.6 (0.2–1.7)	Removed in step-8	48.8	0.7 (0.4–1.2)	Removed in step-7	2.3	0.3 (0.1–1.3)	Removed in step-12
Monthly Income of family									
< = Rs 2020	5.1	1.0	1.0	60.2	1.0	1.0	5.1	1.0	1.0
> Rs 2020	8.2	1.7 (0.6–4.4)	Removed in step-3	51.2	0.7 (0.4–1.1)	Removed in step-10	6.8	1.4 (0.5–3.6)	Removed in step-5
Age of mother (years)									
17–24	6.6	1.0	1.0	50.0	1.0	1.0	5.9	1.0	1.0
25–45	7.4	1.1 (0.5–2.7)	Removed in step-7	57.7	1.4 (0.9–2.1)	1.5 (1.0–2.5)	6.3	1.1 (0.4–2.7)	Removed in step-8
Number of children under five in household									
More than one	6.5	1.0	1.0	53.0	1.0	1.0	5.5	1.0	1.0
Only one child	8.0	1.3 (0.5–2.9)	Removed in step-4	56.8	1.2 (0.7–1.8)	Removed in step-12	7.2	1.3 (0.5–3.3)	Removed in step-4
Consumed milk/its products in last 24 h									
Not consumed	2.5	1.0	1.0	53.8	1.0	1.0	2.5	1.0	1.0
Consumed	14.6	6.6 (2.4–18.3)***	6.2 (2.1–18.6)**	56.1	1.1 (0.7–1.7)	Removed in step-11	12.2	5.3 (1.9–15.1)**	6.2 (2.0–19.0)***

Chi-square *p* values, Yates corrcted: * < 0.05; ** < 0.005, *** < 0.0005

^a^ Only those indicators that were not eliminated from the backward Logistic regression model

## Discussion

Gender equity in childhood nutrition is feasible only by accelerating interventions that aim to improve IYCF practices at a community level. There is growing evidence that IYCF practices are influenced by various social, economic, and cultural factors [16, 30–32]. Findings from an exploratory research study indicate the need to shift focus from nutrition-specific interventions to contextually-appropriate interdisciplinary solutions, incorporating environmental improvements to address the problem of child undernutrition [33]. Our study provides insights on how IYCF practices vary in the context of gender, parental migration, environmental, and contextual factors in the tribal district of Banswara, Rajasthan. Children in this area had relatively good access to immunization services (57%) and access to AWC services (74%), and Ghatol block had access to two crops a year, yet IYCF practices were still found to be sub-optimal. Breastfeeding practices in these areas were better than the dietary practices. In terms of dietary practices, 55% (95% CI: 49–60%) of children received a diet with MMF, while only 7% (95% CI: 5%–11%) received a diet with MDD and 6% (95% CI: 4–10%) received a diet with MAD. Iron-rich food consumption was almost negligible (1.5%;: 95% CI: 0.5–3.6%).

Our study found that male children had significantly better access to a diet with MDD and MAD than the female children – 4.1 times higher for MDD and nearly four times higher for MAD. Gender discrimination in IYCF practices began at infancy, with consumption of each of the seven standard food groups being higher among boys than girls. Children from non-migrant households also had better access to MDD and MAD diets compared to children from migrant households, while the reverse was true for diet with MFF, however, the differences were not statistically significant for all the three key IYCF practices. Migration for livelihood was a common phenomenon in the study population with at least one member (usually a parent, mostly fathers) of the family having migrated for work in the previous year in 46% of households. Children from households without parental migration had higher consumption of milk, eggs, fruit, and vegetables compared to children from households with parental migration. A child from a non-migrant house was 1.9–2.0 times more likely to get a diet with MDD and MAD compared to a child from a migrant house, but this difference was not statistically significant. The insignificant association between migration and MDD and MAD may be because of the huge block-wise variations in migration practices (Kushalgarh-74% Vs Gatol-19%), and the sample size of Kushalgarh block alone was not adequately powered enough to assess this exposure against MDD and MAD.

**Fig. 1:**
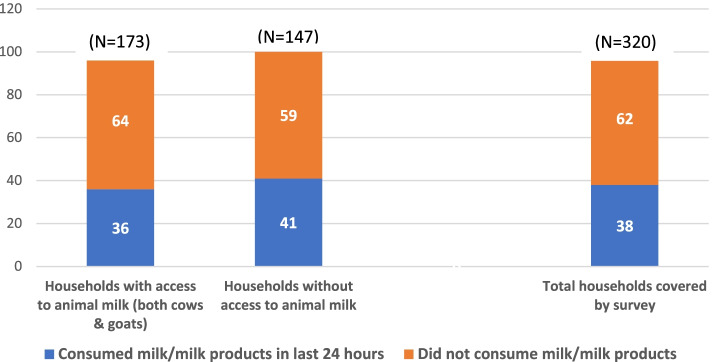
Consumption of milk/milk products according to access to animal milk

Apart from the above two effect modifiers, the two other strongest predictors of improving complementary-feeding practices of MDD and MAD were the presence of milk-producing animals in households and consumption of milk/milk products by children in the previous 24 h. Both these variables were significantly and independently associated with MDD and MAD separately. Children living in households with milk-producing animals had 5.6–5.8 times increased access to a diet with MDD and MAD compared to those living in households without milk-producing animals, while children who had consumed milk/milk products in the previous 24 h had 6 times higher access to MDD and MAD compared to those who did not consume milk/milk products. Overall, a large number of households (60–85%) had milk-producing animals, but less than 40% of children (95% CI: 33.1–44.0) had consumed milk in the previous 24 h. Interestingly, access to animal milk at the household level did not translate to improved consumption of milk or milk products for the children. On the contrary, consumption of milk by children in households with access to animal milk was lower compared to those in households with no/poor access to animal milk (36% vs. 41%) (Fig. 1). One reason for this may be that the majority of households with milk-producing animals sold the milk they got from the domestic animals at the local market to make additional income. This pattern was also observed in the project’s qualitative study [28].

The other significant predictors for improving IYCF practices included literacy of the head of the household (for both MDD and MAD), accessibility of nutrition services at AWC (for MFF only), awareness about the Clean India mission (for MAD only), and use of a clean source of cooking fuel (for MDD only).

### How comparable are our results with other studies?

Two-thirds of the mothers were literate, and 60% of families were aware of the Clean India Mission (a nationwide campaign to eliminate open defecation and improve solid waste management [34]), statistics of this study reflecting the national average. Similarly, study findings also reflected national figures on immunization – 62% of the respondents’ children were fully immunized, 60% received Vitamin supplementation, and 31% were dewormed [13].

According to NFHS-4 (2015–16) in rural Banswara, 39% of children received breastfeeding within 1 hour of birth, 56% were exclusively breastfed, and only 0.8% children aged 6–23 months received MAD. On the other hand, in rural Rajasthan, 29% initiated breastfeeding within 1 hour; 58% exclusively breastfed; 29% of children aged 6–8 months received solid and semi-solid food; and 3.3% received MAD [13]. Most recent findings from CNNS in Rajasthan (2016–18) estimated that while 43.6% of children aged 6–23 months received a diet with MMF, the figures for other complementary feeding indicators were quite low - 11.6% for MDD, 3.5% for MAD and 1.4% for iron-rich foods [12]. Compared to the findings of NFHS-4 and the state CNNS data for both Banswara district and Rajasthan state, the IYCF practices in the PANChSHEEEL study area show improvement (Table 3), but the results are still far from optimal. Additionally, the Banswara district has a higher female-to-male child sex ratio (934) than the state (888) average. Despite this, gender differentials in Banswara district were quite evident on literacy and type of work, with a higher proportion of males being literate (male 70% vs female 43%) and males constituting the main workforce (male 40% vs female 20%), while a higher number of females were marginal workers (male 13% vs female 23%) and agricultural labourers (male 13% vs female 32%) [35]. We also noticed that around one-third of children had consumed convenience foods like biscuits and tea in the previous 24 h. This was also noticed in the formative research, where it was found that when a mother or caregiver is busy with household chores, convenience foods like biscuits, tea, *khichdi* (savoury rice and pulse gruel), *dalia* (porridge with ground wheat with milk or water, mostly sweet) or small pieces of roti dipped in milk are provided to pacify a child’s hunger [28].

An analysis by Fledderjohann showed that breastfeeding patterns were similar for boys and girls until about 12 months of age, when a gender gap begins to emerge. Among the firstborns, the median duration of breastfeeding was around 21 months for females and 23.2 months for males, while second-born females experienced only a slight disadvantage (23.1 months for females and 24.0 for males), highlighting the importance of both gender and birth order in IYCF practices[19].

Based on the variations of type of food consumed and IYCF practices by gender (Table 4), we are unable to provide conclusive evidence that females are discriminated against on all key IYCF practices. However, it is evident that girls were at a disadvantage for most of the complementary-feeding practices compared to the boys, especially access to MDD and MAD. Other studies have reported a lack of conclusive evidence of female children being nutritionally disadvantaged [36] and shown heterogeneity in nutrient intake in different states [37]. One of the reasons for this inconsistency could be because our analysis is aggregated only by gender and not further according to birth order or wealth quintile of children, as these two factors play a key role in gender-based discrimination [37]. A deeper analysis was not possible due to sample size constraints, particularly for MDD and MAD.

Findings from our qualitative formative research in the same nine villages substantiates our finding that girls are at a disadvantage on the education front, particularly those from families with parental migration. In addition, teachers of this area reported that *‘elder siblings, especially girls, were often absent from school to take care of younger siblings, especially in families where both parents have migrated (seasonally) or if primary caregiver was unavailable’*. It was also noticed that, although families do receive food supplements for infants, many mothers don’t know how to prepare them, resulting in children still not getting these benefits [28]. On gender-based labour participation, women were engaged in agriculture and livestock farming throughout the year whilst men took part in agriculture only during sowing and harvesting seasons. Some women with children aged less than 2 years also participated in the National Rural Employment Guarantee Scheme. The men of Ghatol mostly relied on local wage labour due to the proximity of the block to the district headquarters and a cloth mill. Circular migration to urban areas in the adjacent states of Madhya Pradesh and Gujarat was common among men in Kushalgarh.

A study conducted in rural parts of southern Rajasthan (including Banswara district) documented how environmental and contextual factors push families to economic distress, forcing young males to migrate to the neighbouring state of Gujarat for seasonal unskilled jobs [38]. In these migrant households, pressure on financial resources might have led to scarcity of nutritious food and limited the mother’s time and energy that is required to provide adequate care to the young ones. However, in our study we could not find conclusive evidence of parental migration having an independent and statistically-significant effect on a child’s access to key IYCF practices.

### Strengths and limitations

There is limited evidence on the influence of socio-economic factors on IYCF practices [11] and insufficient understanding on the inequalities that shape malnutrition in India [30], particularly in tribal areas [39]. As far as we are aware, this is the first in-depth assessment of inequalities in IYCF practices with respect to gender and parental migration in a tribal district. The study also demonstrates how the factors affecting nutrition for 6–23-month-old children are complex, affected by elements such as gender; poverty and its associated migration; maternal health literacy; home environment; dietary practices; hygiene practices; and access to milk-producing animals. Since data for this study is derived from two divergent blocks of Banswara district and the interventions were co-developed with community stakeholders, findings of this research are applicable to both rural and tribal parts of India.

One of the major limitations of the study is that the findings are based on results from only nine villages and 325 households. Also, the nine villages were selected purposively using a set of inclusion and exclusion criteria, and the eligible households were selected by using a list of lactating mothers made by the AWW/ASHA and not by conducting our own listing and mapping exercise. This approach might have resulted in missing some eligible children from these villages. However, as we adopted a three-step recruitment policy the chances of having missed many eligible households are minimal. Even though there was general improvement in IYCF practices with positive shifts in HEEE factors, most of these associations were not significant/close to significant, perhaps due to limitations of the sample size. Some of the associations between HEEE and IYCF factors have wide confidence intervals due to this limitation. Additionally, this study and other qualitative surveys were conducted during the months January–March 2017, a period of lean agricultural activity, which may have some influence on the IYCF practices.

### Significance

Despite robust programs like ICDS (1975); National Diarrhoeal Diseases Control Programme (1981); National Health Mission & promotion of IYCF (2013); National Deworming Day; Nutritional Rehabilitation Centres (2015); and the Poshan Abhiyaan/National Nutrition Mission (2018), it is evident from our research and recent NFHS-5 (2019–20) findings that the quality of dietary and IYCF practices and the prevalence of child undernutrition is not encouraging [40]. Also, India’s child nutrition issue is characterized by significant inequalities across socioeconomic groups and areas of residence and has made very limited progress in addressing these inequalities.

Although IYCF is generally understood to be shaped by household-level factors, this study emphasizes that IYCF practices are also shaped by contextual factors – especially gender. However, by promoting universal access to animal milk, by engaging with the literate heads of the households in promotion of optimal IYCF, and through effective and targeted implementation of ICDS services, existing gender inequalities in complementary-feeding practices could be minimized. Household-level factors are thus interconnected with the village and HEEE level factors. These should, therefore, be considered when planning an optimum intervention to address IYCF practices in low- and middle-income countries. The challenge of child malnutrition calls for a multidisciplinary approach that targets multiple underlying factors like PANChSHEEEL’s intervention strategy [21], which adopted a multi-disciplinary, participatory, and life-course approach to tackle the multi-dimensional problems of childhood malnutrition and IYCF practices.

## Conclusions

Based on the findings of our research and factsheets of NFHS-5, India’s progress on child undernutrition is not encouraging, barring a few exceptions [40]. With eight out of ten children in India experiencing dietary shortfall and undernutrition as a consequence of several complex factors, and girls and children from parent-migrated homes receiving an inadequate diet, the nutrition agenda of India should have ‘food as a right’ from a holistic perspective. Though efforts have been made to provide affordable access to quality food items for vulnerable households, it is important to urgently address the issue of gender discrimination in dietary practices using integrated and transformative approaches. In addition, efforts need to be made for the provision of adequate and diverse complementary food starting from 6 months old instead of waiting until children reach school age. Such approaches will help India to meet the UN’s SDG targets, working its way towards achieving zero hunger (SDG 2) and good health & well-being (SDG 3).

## Supplementary Information

**Additional file 1.**

**Additional file 2.**

## Data Availability

The data of this study is available from the corresponding author upon reasonable request and data collection tool is provided as attachment.

## Abbreviations

ASHA: Accredited Social Health Activist
AWC: Anganwadi Centre
AWW: Anganwadi worker
BCG: Bacillus Calmette Guerin
CNNS: Comprehensive National Nutrition Survey
DD: Diet Diversity
DPT: Diphtheria, Pertussis, and Tetanus
HEEE: Health, Education, Engineering and Environment
ICDS: Integrated Child Development Services
IRB: Institutional Review Board
IYCF: Infant and Young Child Feeding
MAD: Minimum Acceptable Diet
MCP: Mother and Child Protection
MDD: Minimum Diet Diversity
MMF: Minimum Meal Frequency
NFHS: National Family Health Survey
OPV: Oral Polio Vaccine
PI: Principal Investigator
SDG: Sustainable Development Goal.
ST: Scheduled Tribe
UK: United Kingdom
WHO: World Health Organization
