# Supplementary material for: Influence of gender and parental migration on IYCF practices in 6–23-month-old tribal children in Banswara district, India: findings from the cross-sectional PANChSHEEEL study
Source: BMC Nutr. 2022 Jan 27;8:10. doi: 10.1186/s40795-021-00491-7 (PMC8793254; doi:10.1186/s40795-021-00491-7)
Supplement: Supplementary file 1 — Additional file 1. [file 40795_2021_491_MOESM1_ESM.docx]

| Confidential, information to be used for research purposes only |
| --- |

| **HEEE-MRC Study**  **Household questionnaire** |
| --- |

| **Complete Module 1 -4 by speaking to the head of the household irrespective of the age and sex** | | | | | | | | | | | | | | | | | | |
| --- | --- | --- | --- | --- | --- | --- | --- | --- | --- | --- | --- | --- | --- | --- | --- | --- | --- | --- |
| **Complete Module 5 & 6 by speaking to all the mothers with live child aged less than two years of age**  ***Note: If there is more than one mother with less than 2 years age child, fill module 5&6 for each one of such mothers*** | | | | | | | | | | | | | | | | | |  |
| **Note for Data Collector:** If the house is locked for more than a month, please do **NOT** interview that household. **STOP AND CONFIRM THERE IS NO MEMBER STAYNG IN THE HOUSEHOLD.** | | | | | | | | | | | | | | | | | |  |
| **Form No.** | | | |  |  | |  |  | |  |  |  |  |  |  |  |  |  |
| **IDENTIFICATION** | | | | | | | | | | | | | | | | | |  |
|  | | **NAME** | | | | | | **CODE** | | | **Interview start time**  \|_______\|______\| : \|_____\|______\|  **Hour** **Minute**  **Interview end time**  \|_______\|______\| : \|_____\|______\|  **Hour** **Minute** | | | | | | |  |
| **DISTRICT: ______________** | | | |  | | | |  | | |  |  |  |  |  |  |  |  |
| **BLOCK: ______________** | | | |  | | | |  | | |  |  |  |  |  |  |  |  |
| **VILLAGE : _______________** | | | |  | | | |  | | |  |  |  |  |  |  |  |  |
| **IS THIS A GRAM PANCHAYAT: 1.YES 2.NO** | | | |  | | | |  | | |  |  |  |  |  |  |  |  |
| **IDENTIFIER NUMBER** | | | |  | | | |  | | |  |  |  |  |  |  |  |  |
|  | | | |  | | | |  | | |  |  |  |  |  |  |  |  |
| **INTERVIEWER’S VISITS AND STATUS** | | | | | | | | | | | | | | | | | |  |
|  | **Visit 1** | | | | | **Visit 2** | **Visit 3** | | |  | **Final Visit** | | | | | | |  |
| **Date** | \|_____\|_____\|-\|_____\|_____\|-\|_____\|_____\| | | | | | \|_____\|_____\|-\|_____\|_____\|-\|_____\|_____\| | \|_____\|_____\|-\|_____\|_____\|-\|_____\|_____\| | | |  | **Date** \|_____\|_____\|-\|_____\|_____\|-\|_____\|_____\| | | | | | | |  |
| **Interviewer’s Name** |  | | | | |  |  | | |  | **Interviewer’s code** | | | | \|_____\|_____\|_____\| | | |  |
| **Result code*** | \|_____\|_____\| | | | | | \|_____\|_____\| | \|_____\|_____\| | | |  | **Result code** | | | | \|_____\|_____\| | | |  |
| **Next Visit** | Date: | | | | Date: | |  | | | | **Total # of visits** | | | | \|_____\| | | |  |
|  | Time: | | | | Time: | |  |  |  |  |  |  |  |  |  |  |  |  |
| ***Result Codes:**  01 = Interview completed 02 = Respondent declined interview 03 = Time and date set for later  04 = Respondent not at home 07 = Other, specify: _____________ | | | | | | | | | | | | | | | | | |  |
| **Supervision** | | | **Name** | | | | **Code** | | | | | **Date** | | | | | |  |
| **Checked by SCI supervisor** | | |  | | | | \|____\|____\| | | | | | \|____\|____\|-\|____\|____\|-\|____\|____\| | | | | | |  |
| **Reviewed by QC Team** | | |  | | | | \|____\|____\| | | | | | \|____\|____\|-\|____\|____\|-\|____\|____\| | | | | | |  |
| **Data Entered by** | | |  | | | | \|____\|____\| | | | | | \|____\|____\|-\|____\|____\|-\|____\|____\| | | | | | |  |

# Information For Head of Household And Consent Form

**Instructions to Interviewers:** *Please read and explain the consent to the respondent and answer all questions the respondent may have*

**Study title:** Integrated Health, Education and Environmental (HEE) intervention to optimise infant feeding practices through schools and Anganwadi networks in India

**Approvals:** This study has been approved by UCL Research Ethics Committee: *REF NO: 4032/002* and India-IRB (*REF NO:* 10025/IRB/D/17-18

**Who is conducting the research?**

The study is being sponsored by University College London (UCL) and paid for by the Medical Research Council. Prof Marie-Carine Lall and Dr Lorna Benton from University College London in England and Prof Rajib Dasgupta from Jawaharlal Nehru University in Delhi are conducting this research with colleagues from Save the Children, India. We are visiting 9 villages in the Banswara district of Rajasthan to learn more about how education and engineering can help to improve the nutrition and the health of young children in India.

**Consent**

Namaste. My name is _______. I am working with Save the Children, Rajasthan. We are conducting a survey on influence of health, education, engineering and environment on feeding practices in children under two years of age.

The information on family welfare and feeding practices that we collect from households and individuals will help the government to plan health services. Your household was selected for the survey. I would like to ask you some questions about your household. The questions usually take about 35-45 minutes. All of the answers you give will be confidential and will not be shared with anyone other than members of our survey team. Your participation in the survey is voluntary. If I ask you any question you don't want to answer, just let me know and I will go

on to the next question or you can stop the interview at any time.

If you have any questions about this survey you may ask me.

ANSWER ANY QUESTIONS AND ADDRESS RESPONDENT'S CONCERNS.

If you have any further questions about this survey you may contact the persons listed below:

Name: Dr Hanimi Reddy Modugu (Principal investigator)

Mobile: 99118 22445

Email: [hanimi.modugu@savethechildren.in](mailto:hanimi.modugu@savethechildren.in)

Name: Ms Meena Pahwa (IRB coordinator, Sigma)

Phone: 011-4619 5555

Email: irb.sigma@sigma-india.in

Do you agree to participate in this survey?

May we begin the interview?: Yes🡪 Start interview No🡪 End interview

__________________ (Signature) ­____________ (Date)

Do you need a copy of the consent form? If you want, we can provide you a copy of this form.

***Note:*** *In case the respondent is not able to write, please take written consent from the other family member/neighbors or someone not related to the study. Someone can understand the purpose of the research and the consent form can sign on the consent form.*

**Person obtaining consent**

| RELATION TO INTERVIEWEE | SIGNATURE |
| --- | --- |
|  |  |
|  |  |

In case the respondent is not able to write, please take written consent from the other family member/neighbors or someone not related to the study. Someone can understand the purpose of the research and the consent form can sign on the consent form.

**SECTION 1: HOUSEHOLD MEMBERS DETAILS**

Now we would like some information about the people who usually live in your household or who are staying with you now (Refer to Ration card and/or PDS cards to complete this box quikly) .

| L No | Name  Please give me the names of the  persons who usually live in your  household* and guests of the  household who stayed here last  night, starting with the head of the  household | Sex | Age in completedyears | Relation to head of the house | Marital status | **IF AGE >18 OR OLDER** | | Does  (NAME)  have an  Aadhaar  card? | Eligible for Infant and young child feeding module (under 2 year olds)? | |
| --- | --- | --- | --- | --- | --- | --- | --- | --- | --- | --- |
|  |  |  |  |  |  | Main Occupation | Migrated^#^ for work in past one year? |  | Tick if HH member is under2 years | For each child under 2:  Who is the primary caregiver of  who provides any care for the child, such as feeding, playing or looking after during the day and who is the person that provides most of the care?  **(*NAME*)**?  Record line number of caregiver. |
|  | **Q1** | **Q2** | **Q3** | **Q4** | **Q5** | **Q6** | **Q7** | **Q8** | **Q9** | **Q10** |
| 01 |  |  |  |  |  |  |  |  |  |  |
| 02 |  |  |  |  |  |  |  |  |  |  |
| 03 |  |  |  |  |  |  |  |  |  |  |
| 04 |  |  |  |  |  |  |  |  |  |  |
| 05 |  |  |  |  |  |  |  |  |  |  |
| 06 |  |  |  |  |  |  |  |  |  |  |
| 07 |  |  |  |  |  |  |  |  |  |  |
| 08 |  |  |  |  |  |  |  |  |  |  |
| 09 |  |  |  |  |  |  |  |  |  |  |
| 10 |  |  |  |  |  |  |  |  |  |  |

*: Generally speaking, persons who are present in the household during the last one month or who are known to be usual residents of the household and have stayed there for part of the past one month or who are not present at the time of visit of the enumerator but are expected to return in a month are treated as ‘usual members’. For the purpose of enumeration the following persons are treated as usual members in a household.

#: Temporary migration to a place that is not intended to be permanent for a specified/limited time period, usually undertaken for employment.

F*or each chi*l*d under 2 years of age, write his/her name,* l*ine number and the name and* l*ine number of his/her primary caregiver (usua*ll*y the mother) in the information pane*l *of the* I*nfant and* Y*oung* C*hi*l*d* F*eeding modu*l*e.*

TOTAL CHILDREN UNDER 2 YEARS OF AGE (from Q9 above) |___|___|

**Module 1: Modified Kuppuswamy’s Socio-economic Scale, 2016***

| **Q. #** | **Question** | **Codes** | **Go to Q** |
| --- | --- | --- | --- |
| 101 | Education of the head of the household | Above Graduation ……….…………….7  Graduation.......………………………………6  Intermediate or diploma ….……………......5  High school certificate ….………………….4  Middle school certificate ….………………...3  Primary school certificate …………………..2  Illiterate……………………………………..1 |  |
| 102 | Occupation of the Head of household | Legislators, Senior Officials & Managers…..10  Professionals……………………….……….9  Technicians and Associate………….……….8  Clerks……………………………….………7  Skilled Workers and Shop & Market Sales worker……………………………….……...6  Skilled Agricultural & Fishery Workers…..….5  Craft & Related Trade Workers………..……4  Plant & Machine Operators and Assemblers..3  Unskilled/Agriculture labourer……………...2  Unemployed…………………………………1 |  |
| 103 | Monthly Family Income in Rs. | ≥ 40,430…………………………………….12  20,210 – 40,429……………………………...10  15,160 – 20,209……………………………….6  10,110 – 15,159……………………………….4  6,060 – 10,109………………………………...3  2,021 – 6,059………………………………….2  ≤ 20,20………………………………………1 |  |
|  | **DON’T ASK: CALCULATE YOUR SELF**  **Based on above codes prepare total score** | **Upper (I)… ..………Score: 26-29…………1**  **Upper Middle (II)….Score: 16-25…….……2**  **Lower Middle (III)…..Score: 11 – 15………3**  **Upper Lower (IV)……Score: 5 -10…………4**  **Lower (V)……………Score < 5……… ..….5** |  |

*: Mahesh R. Khairnar1 & Umesh Wadgave1 & Pranali V. Shim (2017). Kuppuswamy’s Socio-Economic Status Scale: A Revision

of Occupation and Income Criteria for 2016. Indian J Pediatr (January 2017) 84(1):3–6.

Module 2: Literacy profile of household members

| **COPY FROM MODULE 1** | | | | **IF AGE 5 OR OLDER** | | | | | | | **IF AGE IS 5 - 18** | | |
| --- | --- | --- | --- | --- | --- | --- | --- | --- | --- | --- | --- | --- | --- |
| L No | Name  (Start with head of the household) | Sex | Age in completed years | Is (NAME) Literate or non – literate^1^ | Has  (NAME)  ever  attended  school? | What is the  highest  standard  (NAME) has  completed? | Type of Education (Vocational / General/ Professional)^2^ | Has (NAME) dropped out of school or college? | At what standard did he she dropped out? | (DON”T ASK) Based on 5-10 column information – Categorize education level^3^ | Type of school/ college^4^? | Is school / college in village or outside village^5^? | Distance of the school / college from your house? |
| **1** | **2** | **3** | **4** | **5** | **6** | **7** | **8** | **9** | **10** | **11** | **12** | **13** | **14** |
| 01 |  | M F |  | Y N— | Y N— |  | V G P | Y N— |  | a b c d  d e f g | a b | b a— |  |
| 02 |  | M F |  | Y N— | Y N— |  | V G P | Y N— |  | a b c d  d e f g | a b | b a— |  |
| 03 |  | M F |  | Y N— | Y N— |  | V G P | Y N— |  | a b c d  d e f g | a b | b a— |  |
| 04 |  | M F |  | Y N— | Y N— |  | V G P | Y N— |  | a b c d  d e f g | a b | b a— |  |
| 05 |  | M F |  | Y N— | Y N— |  | V G P | Y N— |  | a b c d  d e f g | a b | b a— |  |
| 06 |  | M F |  | Y N— | Y N— |  | V G P | Y N— |  | a b c d  d e f g | a b | b a— |  |
| 07 |  | M F |  | Y N— | Y N— |  | V G P | Y N— |  | a b c d  d e f g | a b | b a— |  |
| 08 |  | M F |  | Y N— | Y N— |  | V G P | Y N— |  | a b c d  d e f g | a b | b a— |  |
| 09 |  | M F |  | Y N— | Y N— |  | V G P | Y N— |  | a b c d  d e f g | a b | b a— |  |
| 10 |  | M F |  | Y N— | Y N— |  | V G P | Y N— |  | a b c d  d e f g | a b | b a— |  |

1: Proxy for literacy: can you read a newspaper and/or can you do receipts or bills (hisaab) – record as literate, otherwise non literate.

2: Vocational: occupation or skill based education (ITI, Polytechnique, etc.) ; General: degree/PG in social sciences; Professional: Engineering, medicine, law, management etc.)

3: a. Primary School; b. Middle School; c. Secondary; d. Higher Secondary; e. Higher Education; f. Technical Education; g. Professional Education

4: a. Government; b. Private

5: a: Within village b: Outside village

| **Q. #** | **Question** | **Codes** | **Go to Q** |
| --- | --- | --- | --- |
|  | **Check columns 9 and 10 of above table and, if any one or more members of the family dropped out, ask the following information** | |  |
| 201 | What do you think are the reasons for stopping studies or dropping out of the school/college by members of the house? | R1:_______________________________  R2:_______________________________  R3: _______________________________ |  |
| 202 | What do you think is the main reason for stopping studies | Main Reason:__________________________ |  |
|  | **Check columns 12-14 and, if any one or more members of the family are attending school/college, ask the following information from the PRIMARY CARE GIVER of the school going child:** | |  |
| 203 | Who in the family looks into education, homework and communicate with teachers of the children attending school? | Write relationship of primary care giver to school going child:____________________________  Child’s Mother…………………………….1  Child’s Father……………………………….2  Child’s Grand Parents………………………3  Brother/sister……………………………….4  Other family members/relatives…………….5  No one looks into education………………...8 |  |
| 204 | Do elder children support family in household employment or agriculture? | Yes…………………………………………...1  No……………………………………………2 |  |
| 205 | Do elder children take care of smaller siblings? | Yes………………………………………….1  No………………………………..…………2 |  |
| 206 | How often do parents and other close relatives like grand parents/elder siblings of school going child visit school/ college and keep relationship with teacher(s)? | Once in a week…………………………….1  Once in a month…………………………...2  Rarely……………………………..….…….3  Never……………………………..………..4 |  |
| 207 | Does primary care giver supervise child’s homework? | Yes…………………………………………1  No…………………………………………2 |  |
| 208 | Does parents and other close relatives like grand parents/elder siblings of school going child attend school on parent teacher day? | Yes…………………………………………..1  No……………………………………………2 |  |
| 209 | What language is spoken by teachers while communicating with parents?  WRITE LANGUAGE: ____________________ | Hindi………………………………………1  Wagdi………………………………………2  Other………………………………………9 |  |
| 210 | Do parents teacher’s meeting happen in the school/ college attended by your child? | Yes…………………………………………1  No…………………………………………2  Not aware of such meetings………………..3 | ->212  ->212 |
| 211 | If meetings happen, who from your family attends such meetings? | Write relationship of primary care giver to school going child:____________________________  Child’s Mother…………………………….1  Child’s Father……………………………….2  Child’s Grand Parents………………………3  Brother/sister……………………………….4  Other family members/relatives…………….5  No one looks into education………………...8 |  |
| 212 | Does the parent or caregiver of child regularly enquires about the education/performance of child? | Yes…………………………………………1  No…………………………………………2  Does not enquire………….………………..3 |  |

Module 3: Other socio-demographic characteristics of family

| **Q. #** | **Question** | **Codes** | **Go to Q** |
| --- | --- | --- | --- |
| 301 | What is your Religion? | Hindu 1  Muslim 2  Christan 3  Jain 4  Buddhist 5  Sikh 6  No Religion 7  Other (specify)…………………….. 98 |  |
| 302 | What is your caste?  _______________________________________  ***RECORD AS STATED BY RESPONDENT*** | Scheduled Tribe 1  Scheduled Caste 2  OBC 3  General 4  Other (Specify)……………………………….98 | 304 |
| 303 | If Scheduled Tribe, what is your tribal status? | Name of tribe:__________________________ |  |
| 304 | What language is used for communication in the house?  _______________________________________  ***RECORD AS STATED BY RESPONDENT*** | Hindi 1  Wagdi 2  Other (Specify)……………………………….98 |  |
| 305 | How much total land do members of this household own? | Bigas: \|____\|____\|\|____\|. !___! |  |
| 306 | How much of total land is agricultural land (irrigated and non-irrigated)?  _______________________________  (IF NOT IN ACRES, SPECIFY SIZE AND UNIT) | Bigas \|____\|____\|\|____\|. !___! |  |
| 307 | Out of the Total agriculture land how much is irrigated?  _______________________________  (IF NOT IN ACRES, SPECIFY SIZE AND UNIT) | Bigas \|____\|____\|\|____\|. !___! |  |
| 308 | Does your household own any of the following animals? | Cows, bulls, or buffaloes.…………..Y……..N  Horses, donkeys, or mules…………Y……..N  Goats...…………………………….Y……..N  Sheep....……………………………Y……..N  Chickens or ducks...………………...Y….….N  Other (specify)......…………………..Y…….N | If all are ‘NO’ skip to 312a |
| 309 | Does these anaimals stay in the same dwelling unit or kept in a separate place? | Under same roof………………………..1  Under Separate roof…………………………2 |  |
| 310 | What is done with the produce from anaimals like milk, butter, curd etc? | Only consumed in house…………………1  Only sold outside house…………………..2  Both consumed in house and sold outside….3 | 🡪312  🡪312 |
| 311 | If both, what proportion or percent of the milk/butter etc is sold outside house? | PERCENT \|____\|____\|\|____\| |  |
| 312 | **Check for goats/sheep/chickens/ducks etc…**  What is done with above animals nurtured in the house? | Only consumed in house…………………1  Only sold outside house…………………..2  Both consumed in house and sold outside….3 |  |
| 312a | When members of your household get sick with simple cold/caugh or fever, where do they generally **First** go for treatment?  Write Place:______________________ | **Government:**  Medical college……………………………...1  District Hospital.…………………………..2  CHC/SDH……………..…………………3  PHC………………………………………4  HSC/ANM….…………………………….5  ASHA……………………………………..6  **Other than Government:**  Private clinic/hospital/Dispensary………...7  Private AYUSH clinic/doctor…………….8  Village RMP/Quack……………………….9  Other (Specify)……………………………98 | Q314 |
| 313 | Why don't members of your household generally go to a **government facility** when they are sick?  Any other reason?  RECORD ALL MENTIONED. | No nearby facility/long distance. . . . . …. . . . A  Lack of proper transport…………………….B  Facility opening times not convenient… . . . . C  Health personnel often absent……………. . D  Waiting time too long. ……... …. . . . . . . . . . E  Poor quality of care………. … . . . . . . . . . . . F  Other (specify) ……………………………...Y |  |
| 314 | When 0-3 year children get sick due to Pneuminia/ Diarrhoea, where do you generally go **First** for treatment?  Write Place:______________________ | **Government:**  Medical college……………………………...1  District Hospital.…………………………..2  CHC/SDH………………………3  PHC………………………………………4  HSC/ANM….…………………………….5  **Other than Government:**  Private clinic/hospital/Dispensary………...6  Private AYUSH clinic/doctor…………….7  Village RMP/Quack……………………….8  Other (Specify)……………………………98 |  |
| 315 | During the past one month did any community health worker (CHW) visited your house to advice on child play / development? | Yes 1  No…………………………………………2 | 🡪317 |
| 316 | Who visited? | ASHA……………………………………….1  ANM………………………………………..2  AWW………………………………………..3  Other (specify)………………………………9 |  |
| 317 | Does any usual member of this household have a bank account or a post office account? | Yes 1  No……………………………………………2 |  |
| 318 | Is any usual member of this household covered by a health insurance? | Yes 1  No……………………………………………2 | 🡪320 |
| 319 | What type of health scheme or health insurance?  MULTIPLE RESPONSES | Type of Insurance:______________________  Type of Insurance:______________________ |  |
| 320 | Does your family has a Ration card? | Yes 1  No……………………………………………2 | 🡪323 |
| 320a | ASK FAMILY TO SHARE THE CRAD  Note down the color/category of the card? | Color/Category:____________________ |  |
| 320b | ASK FAMILY TO SHARE THE CRAD  Note down how many members of the family are included in the Ration card? | Members in card:____________________ |  |
| 320c | Does your household get any ration/food using that Ration card from Public Distribution System (PDS)? | Yes 1  No……………………………………………2 | 🡪323 |
| 321 | How frequently do you get ration? | Monthly…………………………………..1  Quarterly………………………………….2  Irregulalry…………………………………3  Only at the time of festivals………………..4  Other (specify)……………………………..8 |  |
| 322 | What type of food/ ration do you get from PDS? | Type of food Quantity  1:_____________ _________  2: ___________ __________  3: __________ ____________  4:__________ ____________ |  |
| 323 | Did any member of this household worked under MNREGA? | Yes 1  No……………………………………………2 | 🡪 326 |
| 324 | If yes, how many members? | How many………………………… |  |
| 325 | Total days of employment got by all the members of the house in last one year? | Days……………..……………… |  |
| 326 | Does this household has a BPL card? | Yes 1  No……………………………………………2 |  |
| 327 | Do you cultivate a ‘kitchen garden’ in your house? | Yes 1  No……………………………………………2 | 🡪331 |
| 328 | If yes, what vegetables/fruits did you grow in that kitchen garden in last year? | Type Grown: ________________________ |  |
| 329 | If yes, where do you get the water to cultivate this garden from? | Within the house, waste water………………1  Within the house, fresh water given………...2  From water source outside home…………...3  Only rain water……………………………..4  Other (specify)……………………………..9 |  |
| 330 | How do you use the vegitables/fruits grown in your kitchen garden? | Only for household consumption…………1  Only for selling in market………………….2  Both of above………………………………3 |  |
| 331 | Does any member(s) of your household migrated in last one year? | Yes 1  No……………………………………………2 | 🡪334 |
| 332 | Number of times migrated? | Times……………..……………… |  |
| 333 | Migrated with… | Only head of the household…………………1  With mother………………………………...2  Without mother…………………………….3  With children……………………………….4  Without children……………………………5  Other (specify)……………………………...9 |  |
| 334 | Does your household have……: | YES NO  A pressure cooker? 1 2  A table? 1 2  A chair? 1 2  Windows with glass? 1 2  A mattress? 1 2 |  |

Module 4: Engineering and Environment

| **Q. #** | **Question** | | **Codes** | | **Go to Q** |
| --- | --- | --- | --- | --- | --- |
|  | **WATER** | | | |  |
| 401 | What is the main source of drinking water for members of your househould? | | Piped water (Dwelling/Yard/Plot) 1  Piped water (Public Tab) 2  Hand Pump into Dwelling/Yard/Plot 3  Public Hand Pump/Tube well/Borehole 4  Covered Dug Well 5  Uncovered Dug Well 6  Rainwater 7  Surface water (river/dam/lake/pond/stream)..8  Other (Specify) 98 | |  |
| 402 | Where is the above water source located? | | In own dwelling . . . . . . . . . . . . . . . . . 1  Elsewhere . . . . . . . . . . . . . . . … . . . . 2  If Elsewhere, how long does it take to reach water source in Minutes . .. . . . \|____\|____\|\|____\| | |  |
| 403 | If there's a piped connection/hand pump within house, the amount of time it takes to fill a standard bucket of 20 liters | | Minutes . .. . . . . . . . . \|____\|____\| | |  |
| 404 | Is water from piped connection or tube well within house, is water available from the source throught the year? | | Yes………………………………………..1  No……………………………….………2 | | 🡪406 |
| 404a | If No, for how many months water is not available in a year? | | Months . .. . . . . . . . . \|____\|____\| | |  |
| 405 | Who usually goes to fetch water for bathing and cleaning utensils in your household? | | Adult woman ≥15 years. . . . . . . . . . . . . . . 1  Adult man .≥ 15 years. . . . . . . . . . . . . . . . . 2  Female child Under age 15 years . . ……. . 3  Male child under age 15 years . . . …... . . . 4 | |  |
| 406 | How much time does it take to fetch water for bathing and cleaning utensils for the household in a day? | | Minutes . .. . . . . . . . . \|____\|____\|\|____\| | |  |
| 407 | How many times in a day do you fetch water for bathing and cleaning utinicels? | | Timess . .. . . . ……….. . . . . \|____\|____\| | |  |
| 408 | Where do you generally store drinking water? | | Pots……………………………………..1  Cement blocks…………………………..2  Plastic buckets…………………………..3  Other (specify)…………………………..9 | |  |
| 409 | How much water do you use for drinking in summer? | | Range:____________ | |  |
| 410 | How much water do you use for cooking, washing, bathing in terms of standard buckets of 20 liters? | | Buckets:_________ | |  |
| 411 | How much do you spend on total water use in house in a year? | | Rs . .. . . . . . . . . \|____\|____\|\|____\| | |  |
| 412 | Does this household do anything to the water to make it safer to drink? | | Yes . . . . . . . . . . . . . . . . . . . . . . . . . . . . . . 1  No . . . . . . . . . . . . . . . . . . . . . . . . .. . . . . . 2  Don't know . . . . . . . . . . . . . . . . . . . . ……8 | | 🡪414  🡪414 |
| 413 | What does this household usually do to make the water safer to drink?  Anything else?  RECORD ALL MENTIONED. | | Boil . . . . . . . . . . . . ………. . . . . . . . . . . . . . . . A  Use alum . . . . . . . . . ………. . . . . . . . . . . . . . . B  Add bleach/chlorine tablets ………………... C  Strain through a cloth …………….. . . . . . . . D  Use water filter (ceramic/  Sand/composite/etc.) ………. …….. . . . . . . E  Use electronic purifier……… . …….. . . . . . . F  Record all mentioned. Let it stand and settle . G  Other (specify)..……………………………X  Don't know ………. . . . . . . . . . . . . . . . . . . . . Z | |  |
| 414 | What do you do with water from cooking, bathing, washing? | | Throw in yard………………………………1  Reuse for growing vegetables……………….2  Leave it to dry by it self……………………..3  Throw in gutter……………………………..4  Other(specify)………………………………9 | |  |
| 415 | Where do households wash their hand? | | Specify:_________________________  No specific place………………………….8 | |  |
| 416 | OBSERVE ONLY  Observe Preseence of water at the place of hand washing | | Water is available……………………………1  Water is not available………………………..2 | |  |
| 417 | OBSERVE ONLY  Observe Preseence of Soap, ash, detergent | | Soap or Detergent…………………………..1  Mud or Ash or Sand………………………...2  None………………………………………..3 | |  |
|  | **SANITATION** | | | |  |
| 418 | Do you have toilet in house or yard? | | Yes, in use. . . . . . . . . . . . . . . . . . . . . . . . . . . 1  Yes, not in use…………………………….2  No . . . . . . . . . . . . . . . . . . . . ….. . . . .. . . . . . 3 | | 🡪423 |
| 419 | What kind of toilet facility do member of your household usually use? | | **Flush or pour flush toilet**  Flush to piped sewer System…………………11  Flush to septic tank……………….. . . . . . . . . 12  Flush to pit latrine ………………. . . . . . . . . . 13  Flush to somewhere else……………….. . . . . 14  Flush, don't know where ……………….. . . . 15  **Pit latrine**  Ventilated improved Pit (vip)/biogas latrine. . 21  Pit latrine with slab……………….. . . . . . . . . 22  Pit latrine without slab/ Open pit . . . . . . . . . . 23  Twin pit/composting toilet…………. . . . . . . 31  Dry toilet …………. . . . . . . . . . . . . . . . . . . . . 41  Uses open space Or field ………………. . . . 51  Other (specify)……………………………….98 | |  |
| 420 | Do you have light inside the toilet for night time use? | | Yes . . . . . . . . . . . . . . . . . . . . . . . . . . . . . . 1  No . . . . . . . . . . . . . . . . . . . . . . . . .. . . . . . 2 | |  |
| 421 | Do you share this toilet facility with other households? | | Yes . . . . . . . . . . . . . . . . . . . . . . . . . . . . . . 1  No . . . . . . . . . . . . . . . . . . . . . . . . .. . . . . . 2 | | 🡪424 |
| 422 | How many households use this toilet facility? | | No. Of households If less than ten  Ten or more households . . ….. . . . . . 95  Don't know . . . . . . . . . . . . . . . . . . . . . 98 | |  |
| 423 | If no Toilet in house, then what facility do you use? | | Public/communal toilet……………………1  Open defecation in own yard………………2  Open defecation in common field…………3  Open defecation in lake/river/steam………4  Other (specify)……………………………..9 | |  |
| 424 | How much do you spend on sanitation in house per month? | | Rs . .. . . . . . . . . \|____\|____\|\|____\| | |  |
|  | **ENERGY** | | | |  |
| 425 | What type of fuel does your household **mainly** use for cooking food? | | Electricity 1  LPG/Natural Gas 2  Bio Gas 3  Kerosen 4  Coal/Light 5  Charcoal 6  Wood 7  Strow/Shrubs/Grass 8  Agricultural Crop Waste 9  Dung Cakes 10  Other (Specify)………………….…………..98 | |  |
| 426 | Cost incurred to family on cooking fuel for one month? | | Rs . .. . . . . . . . . \|____\|____\|\|____\| | |  |
| 427 | Do you have a separate room which is used as a kitchen? | | Yes . . . . . . . . . . . . . . . . . . . . . . . . . . . . . . 1  No . . . . . . . . . . . . . . . . . . . . . . . . .. . . . . . 2 | |  |
| 428 | What type of fuel does your household **mainly** use for lighting? | | Electricity…………………………………..1  Kerosene lamp……………………………..2  Candle……………………………………..3  Solar light………………………………….4  Other (specify)…………………………….9 | | 🡪435 |
| 429 | What electrical equipment do you use in house? | | Radio……………………………Y N  Mobile………………………… Y N  Television…………………… Y N  Fridge……………………… Y N  Fan…………………………… Y N  Other (specify)………………… Y N | |  |
| 430 | What was the total cost for electricity in the household over the last one month? | | Rs . .. . . . . . . . . \|____\|____\|\|____\| | |  |
| 431 | Do you have power cuts? | | Yes . . . . . . . . . . . . . . . . . . . . . . . . . . . . . . 1  No . . . . . . . . . . . . . . . . . . . . . . . . .. . . . . . 2 | |  |
| 432 | During power cut season, generally, for how many hours in a day there is no electricity? | | Hours ………... .. . . . . . . . . \|____\|____\| | |  |
|  | **HOUSE MATERIAL** | | | |  |
| 433 | What is the main material that the Floor of your household is made of?  RECORD, DON’T ASK | | Kutcha………………………………………1  Semi Pucca…………………………………..2  Pucca………………………………………...3 | |  |
| 434 | What is the main material that the Roof of your household are made of?  RECORD, DON’T ASK | | Kutcha………………………………………1  Semi Pucca…………………………………..2  Pucca………………………………………...3 | |  |
| 435 | What is the main material that the Walls of your household are made of?  RECORD, DON’T ASK | | Kutcha………………………………………1  Semi Pucca…………………………………..2  Pucca………………………………………...3 | |  |
|  | **FLOODING** | | | |  |
| 436 | Did your house got flodded in last (August 2017) floods? | | Yes . . . . . . . . . . . . . . . . . . . . . . . . . . . . . . 1  No . . . . . . . . . . . . . . . . . . . . . . . . .. . . . . . 2 | | 🡪440 |
| 437 | If yes, for how many days and how many times last year | | Days…………………………….\|____\|____\|  Times……..…………………….\|____\|____\| | |  |
| 438 | Did your house got damaged by rain in last floods? | | Yes . . . . . . . . . . . . . . . . . . . . . . . . . . . . . . 1  No . . . . . . . . . . . . . . . . . . . . . . . . .. . . . . . 2 | |  |
| 439 | If yes, what does it cost to repair the house | | Rs. . . . . . . \|____\|____\|\|____\|\|____\|\|____\| | |  |
| 440 | Did the main road got flooded in last rainy season | | Yes . . . . . . . . . . . . . . . . . . . . . . . . . . . . . . 1  No . . . . . . . . . . . . . . . . . . . . . . . . .. . . . . . 2 | |  |
| 441 | Due to floods in last year for How many days your children cannot go to school | | Days…………………………….\|____\|____\|  Not Applicable…………………..99 | |  |
|  | **SOLID WASTE** | | | |  |
| 442 | Where do the household throw kitchen waste? | | Back yard of house……………………………1  Main road…………………………………….2  Open plot outside house……………………..3  Use it as gober………………………………..4  Mix with gober……………………………….5  Other (please specify)…………………………9 | |  |
| 443 | How is solid waste in village removed? | | Specify:_______________________________ | |  |
| 444 | Do you practice composting agriculture waste? | | Yes . . . . . . . . . . . . . . . . . . . . . . . . . . . . . . 1  No . . . . . . . . . . . . . . . . . . . . . . . . .. . . . . . 2 | |  |
| 444a | Have you heard/read/seen about “Swatchha Bahrat’ campaign, informing you about how to keep your surroundings clean? | | Yes . . . . . . . . . . . . . . . . . . . . . . . . . . . . . . 1  No . . . . . . . . . . . . . . . . . . . . . . . . .. . . . . . 2 | |  |
| 445 | Have you seen/heard/read message on **Personal Hygiene or cleanliness or use of toilets ?**  **(Multiple responses)** | YES …1  NO….2 | | Television……………………..A  Radio…………………………B  Newspaper/book/magagine….C  Drama/song/nukkad natak…..D  Exhibition/Mela……………...E  Group meeting/Programs……F  Doctor/ASHA/ANM/AWW…G  Friends/relatives………………H  Other (specify)………………...X |  |
| 446 | Have you seen/heard/read the messages on **Safe drinking water?** | YES …1  NO….2 | | Television……………………..A  Radio…………………………B  Newspaper/book/magagine….C  Drama/song/nukkad natak…..D  Exhibition/Mela……………...E  Group meeting/Programs……F  Doctor/ASHA/ANM/AWW…G  Friends/relatives………………H  Other (specify)………………...X |  |

Module 5: Infant and Young Child Feeding (IYCF)

(Only for mothers with children less than two years)

| **Q. #** | | | **Question** | | | | | | **Codes** | | | | | | | | | | | **Go to Q** | | |
| --- | --- | --- | --- | --- | --- | --- | --- | --- | --- | --- | --- | --- | --- | --- | --- | --- | --- | --- | --- | --- | --- | --- |
|  | | | **INFORMATION PANEL** *(*t*his information is entered after identifying eligible children from the* h*ousehold* r*oster)*  Name of child*(from co*l*umn 2 of househo*l*d* r*oster)*: ___________________________________  Sex of child*(from co*l*umn 3 of househo*l*d roster)* (1=male; 2=female): \|___\|  Nameofcaregiver*(from co*l*umn 1 of househo*l*d* r*oster)*: _____________________________________  ***************  **This module is to be administered to the caregiver (USUALLY The mother) of child recorded in the household roster as less than two years of age.**  **Aseparate module should be completed for each eligible child.**  ******************  *Verify that you are speaking with the correct respondent by:*  *1. checking that the respondent’s name is the same as the name of caregiver* l*isted in the information pane*l*above.*  *2. checking that the respondent is the primary caregiver (which is usua*ll*y the mother) of* ***(NAME)***.  I*f the person you are speaking* WITHIS*not that individua*l*, ask to speak with the correct respondent.* | | | | | | | | | | | | | | | | |  | | |
| 501 | | | I would like to ask you some questions about ***(***NAME***)***.  In what month and year was ***(***NAME***)*** born?  What is his/her birthday?  *If the respondent does not know the exact birthdate ask:*  Does ***(***NAME***)*** have a health/vaccination card with the birthdate recorded?  I*f the hea*l*th/vaccination card is shown and the respondent confirms the information is correct, record the date of birth as documented on the card.* | | | | | | DAY.......................................... \|___\|___\|  *If day is not known, enter ‘98’*  MONTH.................................... \|___\|___\|  YEAR.................... \|___\|___\|\|___\|___\| | | | | | | | | | | |  | | |
| 502 | | | How old was (NAME) at his/her last birthday?  R*ecord age in comp*l*eted years.* | | | | | | Lessthan1 year.................................... 0  1-2 years.................................................... 1 | | | | | | | | | | |  | | |
| 503 | | | How many months old is (NAME)?  R*ecord age in comp*l*eted months.* | | | | | | Age in completed months ...... \|___\|___\| | | | | | | | | | | |  | | |
| 504 | | | C*heck* q*uestions 501, 502 and 503 to verify consistency*  *a)* I*s the year recorded in Q501 consistent with age in years recorded in Q502?*  *b)* A*re year and month of birth recorded in Q501 consistent with age in months recorded in Q503?*  IF*the answer to 504a or 504b is ‘*N*o’, reso*l*ve any inconsistencies.* I*f the birthdate was recorded* ON*a hea*l*th card, this may be used as the correct data source.* | | | | | | Consistent with Q502:  YES..........................................................1 NO............................................................ 2  Consistent with Q503:  YES.......................................................... 1 NO............................................................ 2 | | | | | | | | | | |  | | |
| 505 | | | *CHECKcQUESTION 503.*  *IS THE CHILD LESS THAN 24 MONTHS?* | | | | | | YES.......................................................... 1  NO............................................................ 2  DON’TKNOW ........................................... 8 | | | | | | | | | | | ➞ END  ➞ END | | |
| 506 | | | Has ***(***NAME***)*** ever been breastfed? | | | | | | YES.......................................................... 1  NO............................................................ 2  DON’TKNOW ........................................... 8 | | | | | | | | | | | ➞ 507a  ➞ 507a | | |
| 506a | | | After delivery when (NAME) was initiated breastfeeding? | | | | | | < one hour………………………………1  1-24 hours………………….……………2  >24 hours………………..………………3 | | | | | | | | | | |  | | |
| 506b | | | **If the child is more than six months:**  Did you feed any thing like water/honey/gutti etc any time during first six months? | | | | | | Only fed breast milk…………………….1  Fed other things with breast milk………...2  Don’t remember…………………………3 | | | | | | | | | | |  | | |
| 507 | | | Was ***(***NAME***)*** breastfed yesterday during the day or at night? | | | | | | YES.......................................................... 1  NO............................................................ 2  DON’TKNOW ........................................... 8 | | | | | | | | | | | ➞ 508 | | |
| 507a | | | Sometimes babies are fed breast milk in different ways, for example by spoon, cup or bottle. This can happen when the mother cannot always be with her baby. Sometimes babies are breastfed by another woman, or given breast milk from another woman by spoon, cup or bottle or some other way. This can happen if a mother cannot breastfeed her own baby.  Did ***(***NAME***)*** consume breast milk in any of these ways yesterday during the day or at night? | | | | | | YES.......................................................... 1  NO............................................................ 2  DON’TKNOW ........................................... 8 | | | | | | | | | | |  | | |
| 508 | | | Now I would like to ask you about some medicines that are sometimes given to infants.  Was ***(***NAME***)*** given any medicines as drops yesterday during the day or at night? | | | | | | YES.......................................................... 1  NO............................................................ 2  DON’TKNOW ........................................... 8 | | | | | | | | | | |  | | |
| 509 | | | Was ***(***NAME***)*** given [*LOCAL NAME FOR ORS*] yesterday during the day or at night? | | | | | | YES.......................................................... 1  NO............................................................ 2  DON’TKNOW ........................................... 8 | | | | | | | | | | |  | | |
|  | | | R*ead the* q*uestions be*l*ow.* R*ead the* l*ist of* l*i*q*uids one by one and mark yes or no,* ACCORDING*L*Y*.* A*fter you have comp*l*eted the* l*ist, continue by asking* q*uestion 511 (see far right hand co*l*umn) for those* ITEMS*(510*B*, 510*C*,* A*nd/or 510f) where the respondent rep*l*ied ‘yes’.* | | | | | | | | | | | | | | | | |  | | |
| 510 | | |  | Questions and Filters | | | Coding categories | | | | | | | | Questions and codingcategories | | | | |  | | |
|  |  |  |  | Next I would like to ask you about some liquids that ***(***NAME***)*** may have had yesterday during the day or at night.  Did ***(***NAME***)*** have any ***(***ITEM FROM LIST***)***?:  R*ead the* l*ist of* l*i*q*uids starting with ‘p*l*ain water’.* | | |  | | YES | NO | | | DK | 511 How many times yesterday during the day or at night did ***(***NAME***)*** consume any ***(I***TEM FROM LIST***)***?:  *Read Question 511 for items* B*,* C*, and* F*if chi*l*d consumed the item.*  R*ecord ‘98’ for* D*on’t* K*now*. | | | | |  | | |  |
|  |  |  | A | Plain water? | | | A:_____ | |  |  | | |  |  | | | | |  |  |  |  |
|  |  |  | B | Infant formula such as Janamgutti or grypewater | | | B:_____ | |  |  | | |  | No of times: \|___\|___\| | | | | |  |  |  |  |
|  |  |  | C | Milk such as tinned, powdered, or fresh animal milk | | | C:_____ | |  |  | | |  | No of times:\|___\|___\| | | | | |  |  |  |  |
|  |  |  | D | Juice or juice drinks? | | | D:_____ | |  |  | | |  |  | | | | |  |  |  |  |
|  |  |  | E | Clear broth? | | | E:_____ | |  |  | | |  |  | | | | |  |  |  |  |
|  |  |  | F | Yogurt? | | | F:_____ | |  |  | | |  | No of times: \|___\|___\| | | | | |  |  |  |  |
|  |  |  | G | Thin Porridge? | | | G:_____ | |  |  | | |  |  | | | | |  |  |  |  |
|  |  |  | H | Any other liquids such as [**list other water-based liquids available in the local setting**]? | | | H:_____ | |  |  | | |  |  | | | | |  |  |  |  |
|  |  |  | I | Any other liquids? | | | I:_____ | |  |  | | |  |  | | | | |  |  |  |  |
| 512 | | | Do you provide non-vegetarian food to your child? | | | | | | YES.......................................................... 1  Some Times.................................................... 2  No........................................... 3 | | | | | | | | | | |  | | |
|  | | |  | | | | | |  | | | | | | | | | | |  | | |
| 514 | | | Dietary Diversity of Children | | | | | | | | | | | | | | | | | | |  |
|  | | | Now I would like to ask you about liquids or foods which *[CHILD’S NAME]* had eaten yesterday (last 24 hours) during the day or at night. Record every item in the box provided below and then interpret the food group whether covered or not by marking “Yes=1” or “No= 2”. | | | | | | | | | | | | | | | | | | |  |
|  | | | Breakfast | | | Snacks | Lunch | | | | | Snacks | | | | | | Dinner | | | |  |
|  | | |  | | |  |  | | | | |  | | | | | |  | | | |  |
| 1 | | | Grains, roots and tubers (all cereals such as rice, wheat, bread, noodles, porridge which are made from grain, roots and tubers like potato, white yam, sweet potato) | | | | | | | | | GRAINS | | | | | Yes= 1, No= 2 | | | | |  |
| 2 | | | Legumes and nuts (Any food made from *Dal* of all kind, beans, peas, lentils, nuts and seeds) | | | | | | | | | LEGUMES | | | | | Yes= 1, No= 2 | | | | |  |
| 3 | | | Diary product, excluding mother’s milk (Animal milk, Yogurt, Cheese, Curd, Infant formula, canned/ powdered milk) | | | | | | | | | MILK | | | | | Yes= 1, No=2 | | | | |  |
| 4 | | | Eggs | | | | | | | | | EGGS | | | | | Yes= 1, No=2 | | | | |  |
| 5 | | | Flesh food (Meat, fish, chicken, Liver, Kidney or any organ meat, beef, pork, lamb, sea food) | | | | | | | | | MEAT | | | | | Yes= 1, No=2 | | | | |  |
| 6 | | | Vit-A reach fruits and vegetables (Pumpkin, carrots, squash or sweet potatoes that are yellow or orange inside, any dark green vegetables like cassava leaves, amaranthus, bean leaves, pumpkin leaves, rape, mustard. Ripe mangos, ripe papaya, musk melon, foods made with red palm oil/ red palm nuts) | | | | | | | | | VITFVEG | | | | | Yes= 1, No=2 | | | | |  |
| 7 | | | Other fruits and vegetables | | | | | | | | | OFRTVEG | | | | | Yes= 1, No=2 | | | | |  |
|  | | |  | | |  |  | | | | |  |  | | | |  |  | | | |  |
| 515 | | Did ***(***NAME***)*** eat any solid, semi-solid, or soft foods yesterday during the day or at night?  *IF‘YES’ PROBE:* What kind of solid, semi-solid, or soft foods did ***(***NAME***)*** eat? | | | | | | | YES.......................................................... 1  GO BACK TO Q12 AND RECORD FOODS *EATEN. THEN CONTINUE WITH Q14*  NO............................................................ 2  DON’TKNOW ........................................... 8 | | | | | | | | | | ➞ 517  ➞ 517 | | |  |
| 516 | | How many times did ***(***NAME***)*** eat solid, semi-solid, or soft foods other than liquids yesterday during the day or at night, including snacks and meals? | | | | | | | NUMBEROFTIMES............... \|___\|___\|  DON’TKNOW ......................................... 98 | | | | | | | | | |  | | |  |
| 517 | | Did ***(***NAME***)*** drink anything from a bottle with a nipple yesterday during the day or night? | | | | | | | YES.......................................................... 1  NO............................................................ 2  DON’TKNOW ........................................... 8 | | | | | | | | | |  | | |  |
| 518 | | Does (NAME) goes to Anganwadi center? | | | | | | | YES.......................................................... 1  NO............................................................ 2  DON’TKNOW ........................................... 8 | | | | | | | | | |  | | |  |
| 519 | | Is (NAME) weiged recently? | | | | | | | YES.......................................................... 1  NO............................................................ 2  DON’TKNOW ........................................... 8 | | | | | | | | | | ➞ 601  ➞ 601 | | |  |
| 520 | | Who weighed weight of (NAME)? | | | | | | | AWW……………………………1  ANM……………………………..2  Doctor……………………………3  Other (specify)…………………….8 | | | | | | | | | |  | | |  |
| 521 | | How frequently (NAME) is weighed? | | | | | | | Once in a month……………..……1  Not regularly……………………….2  Don’t know………………………..8 | | | | | | | | | |  | | |  |
| 522 | | What is the most recent weight of (NAME)? | | | | | | | WEIGHT in KG: \|___\|___\|. \|___\| | | | | | | | | | |  | | |  |

Module 6: Immunization and Childhood morbidities management

(Only for mothers with child in the age group of 12-23 months)

| **Q. #** | **Question** | **Codes** | | | | | | | **Go to Q** |
| --- | --- | --- | --- | --- | --- | --- | --- | --- | --- |
| 601 | Within the last six months, was (NAME) given a vitamin A dose ? | Yes . . . . . . . . . . . . . . . . . . . . . . . . . . . . . . 1  No . . . . . . . . . . . . . . . . . . . . . . . . .. . . . . . 2  Don't know . . . . . . . . . . . . . . . . . . . . ……8 | | | | | | |  |
| 602 | Was (NAME) given any drug for intestinal worms in the last six months? | Yes . . . . . . . . . . . . . . . . . . . . . . . . . . . . . . 1  No . . . . . . . . . . . . . . . . . . . . . . . . .. . . . . . 2  Don't know . . . . . . . . . . . . . . . . . . . . ……8 | | | | | | |  |
| 603 | Do you have a card where (NAME'S) vaccinations are written down? IF YES: May I see it please? | Yes, Seen……………………………….1  Yes, Not seen…………………………..2  No card…………………………………3 | | | | | | | 🡪606  🡪606 |
| 604 | Did you ever have a vaccination card for (NAME)? | Yes . . . . . . . . . . . . . . . . . . . . . . . . . . . . . . 1  No . . . . . . . . . . . . . . . . . . . . . . . . .. . . . .2 | | | | | | |  |
| 605 | (1) copy vaccination date for each vaccine from the card.  (2) write ‘44' in ‘day' column if card shows that a vaccination was given, but no date is recorded.  (3) if only part of date is shown on card, record '98' for 'don't know' in the column for which information is not given. |  | DD | MM | YY | Mother reported | Not received |  |  |
|  |  | BCG |  |  |  |  |  |  |  |
|  |  | Polio 0 |  |  |  |  |  |  |  |
|  |  | Polio 1 |  |  |  |  |  |  |  |
|  |  | Polio 2 |  |  |  |  |  |  |  |
|  |  | Polio 3 |  |  |  |  |  |  |  |
|  |  | DPT 1 (PV1) |  |  |  |  |  |  |  |
|  |  | DPT 2 (PV2) |  |  |  |  |  |  |  |
|  |  | DPT 3 (PV3) |  |  |  |  |  |  |  |
|  |  | Hepatitis B0 |  |  |  |  |  |  |  |
|  |  | Hepatitis B1 |  |  |  |  |  |  |  |
|  |  | Hepatitis B2 |  |  |  |  |  |  |  |
|  |  | Hepatitis B3 |  |  |  |  |  |  |  |
|  |  | Measles |  |  |  |  |  |  |  |
|  |  | Vitamin A in last 6 months |  |  |  |  |  |  |  |
|  |  | De-worming in last 6 months |  |  |  |  |  |  |  |
| 606 | Has (NAME) received any vaccinations that are not recorded on this card, including vaccinations received in a Pulse Polio campaign? | Yes . . . . . . . . . . . . . . . . . . . . . . . . . . . . . . 1  No . . . . . . . . . . . . . . . . . . . . . . . . .. . . . . . 2  Don't know . . . . . . . . . . . . . . . . . . . . ……8 | | | | | | |  |
| 607 | Has (NAME) had diarrhoea in the last 2 weeks? | Yes . . . . . . . . . . . . . . . . . . . . . . . . . . . . . . 1  No . . . . . . . . . . . . . . . . . . . . . . . . .. . . . . . 2 | | | | | | | 🡪613 |
| 608 | Was there any blood in the stools? | Yes . . . . . . . . . . . . . . . . . . . . . . . . . . . . . . 1  No . . . . . . . . . . . . . . . . . . . . . . . . .. . . . . . 2 | | | | | | | 🡪613 |
| 609 | Now I would like to know how much (NAME) was given to drink (including breastmilk) during the diarrhoea. Was (he/she) given less than usual to drink, about the same amount, or more than usual to drink? IF LESS, PROBE: Was (he/she) given much less than usual to drink or somewhat less? | Much less ……………………………. . . . . 1  Somewhat less……………………………. . 2  About the same…………………………… . 3  More………………………… . . . . . . . . . . . . 4  Nothing to drink..………………………….. .5  Don't know………………………….. . . . . . 8 | | | | | | |  |
| 610 | When (NAME) had diarrhoea, was (he/she) given less than usual to eat, about the same amount, more than usual, or nothing to eat? IF LESS, PROBE: Was (he/she) given much less than usual to eat or somewhat less? | Much less ……………………………. . . . . 1  Somewhat less……………………………. . 2  About the same…………………………… . 3  More………………………… . . . . . . . . . . . . 4  Nothing to eat..………………………….. .5  Don't know………………………….. . . . . . 8 | | | | | | |  |
| 611 | Did you seek advice or treatment for the diarrhoea from any source? | Yes . . . . . . . . . . . . . . . . . . . . . . . . . . . . . . 1  No . . . . . . . . . . . . . . . . . . . . . . . . .. . . . . . 2 | | | | | | | 🡪613 |
| 612 | Where did you seek advice or treatment?  Anywhere else? | Medical college………………………….A  District Hospital……………………….B  Sub District Hospital…………………..C  HSC/ANM……………………………D  PHC…………………………………...E  APHC………………………………….F  CHC……………………………………G  VHND…………………………………H  AWC……………………………………I  Private clinic/hospital/Dispensary……..J  Village RMP ……………………………K  AYUSH clinic/doctor…………………..L  Other (Specify)………………………X | | | | | | |  |
| 613 | Has (NAME) had an illness with a cough at any time in the last 2 weeks? | Yes . . . . . . . . . . . . . . . . . . . . . . . . . . . . . . 1  No . . . . . . . . . . . . . . . . . . . . . . . . .. . . . . . 2  Don’t Know……………………………8 | | | | | | | 🡪End  🡪End |
| 614 | When (NAME) had an illness with a cough, did (he/she) breathe faster than usual with short, rapid breaths or have difficulty breathing? | Yes . . . . . . . . . . . . . . . . . . . . . . . . . . . . . . 1  No . . . . . . . . . . . . . . . . . . . . . . . . .. . . . . . 2  Don’t Know……………………………8 | | | | | | | 🡪End  🡪End |
| 615 | Was the fast or difficult breathing due to a problem in the chest or to a blocked or runny nose? | Chest Only………………………………1  Nose only………………………………..2  Both……………………………………..3  Other (specify)…………………………..9  Don’t Know……………………………..8 | | | | | | |  |
| 616 | Now I would like to know how much (NAME) was given to drink (including breastmilk) during the above problem. Was (he/she) given less than usual to drink, about the same amount, or more than usual to drink? IF LESS, PROBE: Was (he/she) given much less than usual to drink or somewhat less? | Much less ……………………………. . . . . 1  Somewhat less……………………………. . 2  About the same…………………………… . 3  More………………………… . . . . . . . . . . . . 4  Nothing to drink..………………………….. .5  Don't know………………………….. . . . . . 8 | | | | | | |  |
| 617 | When (NAME) had above problem, was (he/she) given less than usual to eat, about the same amount, more than usual, or nothing to eat? IF LESS, PROBE: Was (he/she) given much less than usual to eat or somewhat less? | Much less ……………………………. . . . . 1  Somewhat less……………………………. . 2  About the same…………………………… . 3  More………………………… . . . . . . . . . . . . 4  Nothing to drink..………………………….. .5  Don't know………………………….. . . . . . 8 | | | | | | |  |
| 618 | Did you seek advice or treatment for above illness (pneumonia) from any source? | Yes . . . . . . . . . . . . . . . . . . . . . . . . . . . . . . 1  No . . . . . . . . . . . . . . . . . . . . . . . . .. . . . . . 2 | | | | | | | 🡪End |
| 619 | Where did you seek advice or treatment?  Anywhere else? | Medical college………………………….A  District Hospital……………………….B  Sub District Hospital…………………..C  HSC/ANM……………………………D  PHC…………………………………...E  APHC………………………………….F  CHC……………………………………G  VHND…………………………………H  AWC……………………………………I  Private clinic/hospital/Dispensary……..J  Village RMP ……………………………K  AYUSH clinic/doctor…………………..L  Other (Specify)………………………X | | | | | | |  |

Thank the respondent for participating in survey
