# Supplementary material for: Influence of gender and parental migration on IYCF practices in 6–23-month-old tribal children in Banswara district, India: findings from the cross-sectional PANChSHEEEL study
Source: BMC Nutr. 2022 Jan 27;8:10. doi: 10.1186/s40795-021-00491-7 (PMC8793254; doi:10.1186/s40795-021-00491-7)
Supplement: Supplementary file 2 — Additional file 2. [file 40795_2021_491_MOESM2_ESM.docx]

Additional file 2

List of Independent variables (background and HEEE characteristics) included in initial analysis and in final regression model

| **S.No** | **Major theme** | **Variable name** | **Definition** | **Coding** | **Included in regression model** |
| --- | --- | --- | --- | --- | --- |
| 1 | Gender | Gender of child | -- | Girl-1  Boy-2 | Yes |
| 2 | Parental migration | Migration | Any adult member/parent of the house migrated for work/extra income outside village in last year | Migrated-1  Not migrated-2 | Yes |
| 3 | Education | Literacy of head of the household | Who can read and write a sentence with understanding | Illiterate-1  Literate-2 | Yes |
| 4 | Education | Literacy of caretaker of child (mother) | Who can read and write a sentence with understanding | Illiterate-1  Literate-2 | Yes |
| 5 | Education | Awareness of head of house about ‘Swachh Bharat Campaign’ | Aware of campaign that promotes personal hygiene and sanitation at individual and community level | Not aware-1  Aware-2 | Yes |
| 6 | Health & Growth monitoring | Access of child to Anganwadi Centre (AWC) | Caretaker reported, index child goes to AWC regularly | No-1  Yes-2 | Yes |
| 7 | Health & Growth monitoring | Child weight monitoring in past three months | Caretaker reported index child’s weight was monitored at AWC monthly, uninterruptedly for the past three months | Not-monitored-0  Monitored-1 | No |
| 8 | Health & Growth monitoring | Child weighed once every month | Caretaker reported index child’s weight is monitored every month | Not-weighed-0  Weighed-1 | No |
| 9 | Health & Growth monitoring | Immunization and/or Mother and Child Protection (MCP card availability | Caretaker showed to investigator either of the cards at the time survey | Not-showed-0  Showed-1 | No |
| 10 | Immunization, Micronutrients & De-worming | Fully immunized children | Child aged 12-23 months, who got one dose of BCG, three injections of DPT, three doses of polio and one injection of measles | Got all-1, Otherwise-0 | No |
| 11 | Immunization, Micronutrients & De-worming | Child who got Vitamin-A | Child aged 12-23 months, who got Vitamin-A supplements in past six months | Did not get-0  Got -1 | No |
| 12 | Immunization, Micronutrients & De-worming | Child de-wormed | Child aged 12-23 months, who got Albendazole tablet in past six months | Did not get-0  Got -1 | No |
| 13 | Engineering & Environment | Houses with kitchen garden | Households who cultivate kitchen garden inside house | Do not cultivate-0  Who cultivate-1 | No |
| 14 | Engineering & Environment | Houses with access to safe drinking water | With access to protected drinking water sources like tube well or covered well water | No-1  Yes-2 | Yes |
| 15 | Engineering & Environment | Houses with improved fuel source | Using gas or electricity for cooking food | No-1  Yes-2 | Yes |
| 16 | Engineering & Environment | Houses with improved sanitation facility | Who use toilet in the house and who wash hands with soap | No-1  Yes-2 | Yes |
| 17 | Milk & Poultry products | Houses with cows/bulls/buffaloes | With one or more cows/bulls / buffaloes – assuming access to animal milk | No-1  Yes-2 | No |
| 17 | Milk & Poultry products | Houses with goats | With one or more goats – assuming access its milk | No-1  Yes-2 | No |
| 18 | Milk & Poultry products | Houses with cows /buffaloes and goats | With one or more cows / buffaloes and goats – assuming access animal milk | No-1  Yes-2 | Yes |
| 19 | Milk & Poultry products | Houses with poultry products | With one or more chickens / ducks – assuming access eggs and their meat | No-1  Yes-2 | No |
| 20 | Milk & Poultry products | Consumed milk/its products | Child consumed milk/milk products in the past 24 hours as reported by mother | No-1  Yes-2 | Yes |
| 21 | Background characteristics | Blocks | -- | Kushalgarh-1,  Ghatol-2 | Yes |
| 22 | Background characteristics | Religion | -- | Other-0  Hindu-1 | No |
| 23 | Background characteristics | Caste | -- | Scheduled tribe-1 Other-2 | Yes |
| 24 | Background characteristics | Monthly family income | Income in Indian rupees INR | ≤2020-1  >2021-2 | Yes |
| 25 | Background characteristics | Main occupation | As specified by head of the household | Cultivation /agriculture-1, Other-2 | No |
| 26 | Background characteristics | Age of mother | Age in years at the time of survey | 25 - 45-0  17 - 24-1 | Yes |
| 27 | Background characteristics | Number of under five children | Number of under five children in house | More than once child-1  Only one child-2, | Yes |
